# Transcriptome assists prognosis of disease severity in respiratory syncytial virus infected infants

**DOI:** 10.1038/srep36603

**Published:** 2016-11-11

**Authors:** Victor L. Jong, Inge M. L. Ahout, Henk-Jan van den Ham, Jop Jans, Fatiha Zaaraoui-Boutahar, Aldert Zomer, Elles Simonetti, Maarten A. Bijl, H. Kim Brand, Wilfred F. J. van IJcken, Marien I. de Jonge, Pieter L. Fraaij, Ronald de Groot, Albert D. M. E. Osterhaus, Marinus J. Eijkemans, Gerben Ferwerda, Arno C. Andeweg

**Affiliations:** 1 Julius Center for Health Sciences and Primary Care, University Medical Center Utrecht, Utrecht, The Netherlands; 2 Department of Viroscience, Erasmus Medical Center, Rotterdam, The Netherlands; 3 Department of Pediatrics, Laboratory of Pediatric Infectious Diseases, Radboud Institute for Molecular Life Sciences, Radboud University Medical Center, Nijmegen, The Netherlands; 4 Center for Biomics, Erasmus Medical Center, Rotterdam, The Netherlands; 5 Department of Pediatrics, Erasmus Medical Center, Rotterdam, The Netherlands; 6 Research Institute for Infectious Diseases and Zoonoses, Veterinary University Hannover, Germany

## Abstract

Respiratory syncytial virus (RSV) causes infections that range from common cold to severe lower respiratory tract infection requiring high-level medical care. Prediction of the course of disease in individual patients remains challenging at the first visit to the pediatric wards and RSV infections may rapidly progress to severe disease. In this study we investigate whether there exists a genomic signature that can accurately predict the course of RSV. We used early blood microarray transcriptome profiles from 39 hospitalized infants that were followed until recovery and of which the level of disease severity was determined retrospectively. Applying support vector machine learning on age by sex standardized transcriptomic data, an 84 gene signature was identified that discriminated hospitalized infants with eventually less severe RSV infection from infants that suffered from most severe RSV disease. This signature yielded an area under the receiver operating characteristic curve (AUC) of 0.966 using leave-one-out cross-validation on the experimental data and an AUC of 0.858 on an independent validation cohort consisting of 53 infants. A combination of the gene signature with age and sex yielded an AUC of 0.971. Thus, the presented signature may serve as the basis to develop a prognostic test to support clinical management of RSV patients.

Respiratory syncytial virus (RSV) causes infections that range from common cold to severe lower respiratory tract infection that in some instances may have a fatal outcome. Especially infants, elderly and patients with underlying chronic disorders suffer from severe RSV infections^1^,^2^. In infants, RSV is the leading cause of lower respiratory tract infections (LRTI) and is responsible for 80% of acute bronchiolitis cases^3^. RSV infections pose a huge burden on society in terms of disease, logistics and socio-economic sequelae. There is an unmet need for an RSV vaccine, despite considerable research efforts no licensed vaccine has been developed.

In industrialized countries, 1–5% of infants with RSV infection are hospitalized^4^,^5^,^6^,^7^. Some of these infants yet suffer from severe disease upon admittance, while others are admitted without severe symptoms since the course of bronchiolitis is highly variable and the need for supportive care cannot be predicted^8^,^9^. Several risk factors for developing severe RSV disease in infants have been identified, including preterm birth, young age, sex and environmental factors like in-house smoking^10^. Notwithstanding these known risk factors, current medical practice does not allow accurate prediction of whether an infant will further progress to severe RSV disease or not and could even be sent home safely. Genomic technologies have contributed to study the virus-host interaction, including virus discovery, pathogenesis studies, the design of antiviral strategies and identification of biomarkers to support clinical management of infectious diseases^11^,^12^,^13^,^14^. For RSV infections, this has supported the characterization of vaccine-induced skewed host responses upon infection^15^,^16^. Meijas *et al*.^17^ recently used blood transcriptome profiles obtained within 3 days of hospitalization to characterize the host response to RSV infection in infants compared with rhinovirus or influenza infections and identified transcriptional profiles that associate with RSV disease severity. However, a prognostic model for RSV severity based on gene expression profiles collected at admittance to the hospital has not been developed.

In this study we aim to identify and validate a gene signature that discriminates severe from less severe RSV LRTI that do not require advanced support. Such a signature together with other clinical parameters may improve the prognosis of less severe patients that could be safely sent home.

## Material and Methods

### Study design

Study subjects were recruited at Canisius Wilhelmina Hospital, Radboud University Medical Center, Nijmegen, and Erasmus Medical Center, Rotterdam, The Netherlands. Nasopharyngeal wash and blood samples were prospectively obtained from patients less than 2 years of age with a viral bronchiolitis. Patient enrolment occurred 7 days a week and samples were taken within 24 hours after first contact with the hospital. Seventy-three percent of all eligible bronchiolitis patients agreed to participate in the study. The major reasons for non-inclusion were parental availability to sign consent and the hesitancy for the venipuncture. Only patients with an RSV infection, as retrospectively determined by PCR were included in the study. Exclusion criteria were: immunodeficiency, systemic steroid treatment in the previous 2 weeks, blood transfusion, congenital heart and chronic lung disease. A Tempus tube (Tempus^TM^, Applied Biosystems, Austria) and sodium heparin tube were filled with 3 ml of blood. Medical history, demographic and clinical data were collected from medical records and questionnaires. The (hospitalized) patients were followed until recovery and were retrospectively classified as: mild for children without hypoxia, moderate for patients requiring supplemental oxygen (oxygen saturations <90%, ≥10 minutes) and severe for children requiring mechanical ventilation due to apnea, exhaustion and/or respiratory failure. Recovery samples were obtained after 4–6 weeks, during home visits. Blood samples were obtained from healthy controls (HC) without underlying diseases or medication subjected to elective surgery.

### Study approval

The study protocol was approved by the Regional Committees on Research involving Human Subjects of Arnhem-Nijmegen and Rotterdam and were conducted in accordance with the principles of the Declaration of Helsinki. Written informed consent was obtained from the parents of all children prior to inclusion in the study.

### Sample processing and blood transcriptome profiling

Inclusion of patients and sample collection was performed by a single MD at the hospitals. Multiplex RT-PCR was performed to test the nasopharyngeal washes on 15 different viral pathogens, as previously described^18^. Blood was collected in Tempus tubes for immediate stabilization of RNA and subsequently stored at −80 °C. Total RNA was isolated from each blood sample, processed, assessed, labelled and hybridized to a single Affymetrix Human Genome U133 plus 2 gene chips; and image analysis was performed in the same lab and by one technician as described in supplementary material. The raw data has been deposited in the ArrayExpress database under access number E-MTAB-5195.

### Data preprocessing

Microarray data was preprocessed using R 3.1.2 19 and Bioconductor^20^. Upon initial quality control and VSN normalization (to render samples comparable), probeset (a combination of multiple probes) summarization was performed by median polish^21^,^22^. Unless otherwise stated, all probesets/genes present on the Affymetrix GeneChip were used for data analysis. Samples were labelled and hybridized in two batches which did not correspond to any biological variable as samples were randomly assigned to the batches. The normalized expression values were adjusted for a batch effect (see supplementary Fig. S1) using *ComBat*^23^. Additionally, we assessed confounding effects of clinical parameters age and sex on gene expression–severity relationship using “biasograms”^24^.

### Differential expression analysis

To obtain a global view of the blood transcriptome changes in response to RSV infection (i.e. to evaluate whether whole transcriptome changes associate with severity), a principal component analysis (PCA) as an exploratory analysis was performed on the age by sex standardized data. Next, a differential expression (DE) analysis was performed on the normalized-batch-adjusted data controlling for an age by sex effect using empirical Bayes linear models^25^ implemented in the R package *limma*^26^. Details of the models are found in supplementary material. We controlled for multiple testing via false discovery rate (FDR) using a Benjamini and Hochberg procedure^27^. Gene set enrichment analysis was performed using Ingenuity pathway analysis (IPA, www.qiagen.com/ingenuity).

### Identification and evaluation of prognostic biomarkers

Since we are interested in identifying RSV-infected infants that will progress to severe stage upon presentation to the hospital, we grouped mild and moderate samples and aimed to separate these samples from infants that were presented with or progressed to severe disease after hospitalization. We chose to utilized probabilistic predictors (to predict the chance of an RSV-infected infant to be severe) because in clinical applications, probabilities are more informative than absolute yes or no predictions^28^. Several probabilistic predictors exist in the literature and their performance depends on the type of the data they are being applied on ref. 29. Using results of ref. 29,30 and observed correlations in the data, three probabilistic classification functions that could be optimal for this data were chosen as described in supplementary material. These functions were support vector machines (SVM)^31^, shrunken centroids discriminant analysis (SCDA)^32^ and random forest (RF)^33^.

For each classification function, the experimental data was split into a learning set and a test set using leave-one-out cross-validation (LOOCV). Cross-validation reduces optimistic bias by ensuring that our models are evaluated on an independent dataset that was not used to constructed these models. Most probabilistic classification functions require hyper-parameters to perform variable selection among the huge number of variables (probesets). Usually, the best values for these hyper-parameters are also determined by cross-validation. Thus, the parameter(s) of the function were optimized using an inner loop of five-fold cross-validation on the learning set. Next, a prognostic model was built with the optimal parameter(s) on the entire learning set and evaluated with the test set, as described in supplementary material. The following R packages; *CMA*^34^*, e1071*^35^, *pamr*^36^ and *randomForest*^37^ were utilized for class prediction. The best calibrated and refined function amongst the three functions was selected and its performance evaluated using the area under the receiver operating characteristic (ROC) curve (AUC). Finally, the transcripts that maximized the binomial log-likelihood function, with the leave-one-out cross-validated data were retained as a gene signature from the selected function as described in supplementary material.

### Comparison of biomarkers to clinical parameters

Age and sex are readily available clinical parameters that have been determined to be associated to RSV disease severity^38^. To assess the gain attained with a genomic model over a model with these clinical parameters, and the effect of standardization, the leave-one-out cross-validated predicted probabilities of progressing to severe for all samples were transformed to genomic scores (a genomic score is single measure of the genome of a sample as predicted by a model) for models with unstandardized and standardized data. Logistic regression models (see supplementary material) were then fitted with the genomic scores and/or clinical parameters and their AUCs compared.

### Validation of biomarkers

For an independent validation, a subset of the Illumina RSV data of Meijas *et al*.^17^ was used. Since the experimental data and validation data were obtained using different platforms, we linked the data using gene symbols and applied cross-platform transformation (to render gene expression comparable across datasets) as described in supplementary material. The transformed data was supplied to our prognostic model for predictions of probabilities of severity. For a confirmatory analysis of how well our prognostic model performs, we built and evaluated a prediction model with the chosen function (same function used to build our prognostic model) on the entire Illumina data and compared our validation performance to the performance from this (unrestricted) data.

## Results

### Study subjects and sampling

Thirty-nine infants hospitalized with acute RSV bronchiolitis were included in the study. Nasopharyngeal wash and whole blood samples for mRNA profiling were collected within 24 hours upon hospital admittance. Table 1 presents the characteristics of the study subjects. As expected, patients with the most severe course of RSV bronchiolitis were significantly younger than those with a relative mild or moderate course of this disease. The variables related to disease severity; duration of oxygen, and length of stay in the hospital were highest in the severe group, with ventilation indicating the method by which oxygen was supplied. The proportion of co-infections was lower in severe patients as compared to the other severity categories. There were no differences in the occurrence of other known risk factors.

### Age and sex as confounders of gene expression–severity relationship

**Figure 1:**
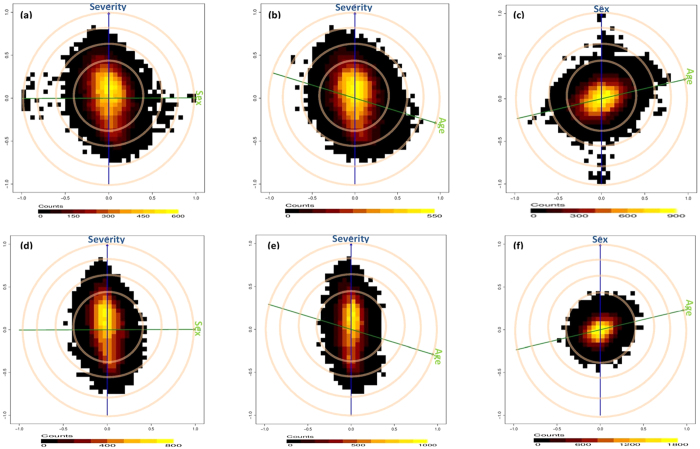
Confounding effect of Sex, Age and Age by Sex on gene expression–severity relationship, before. (**a,b,c**) and after: (**d,e,f**), an age by sex standardization. The blue and green lines represent the clinical variables, the cosine of the angle between the lines represents its correlation to the blue line (Sex is not correlated to Severity, Age is negatively correlated to Severity i.e. younger kids become severe and Age is positively correlated to sex i.e. females are older). The cloud of points represent the transcripts and their correlations to both variables with most transcripts uncorrelated to the variables (yellow cloud) while a considerable number (black cloud) are correlated to Severity, Sex, Age and Age *Sex. The associations between the transcripts and Sex, Age or Age *Sex are significantly eliminated after standardization while retaining that of Severity.

Figure 1a,b respectively illustrate the confounding effects of sex and age on the gene expression-disease severity relationship. These figures show that whereas age is negatively correlated to severity, sex is uncorrelated to severity. Nevertheless, the high positive/negative correlations of a considerable number of transcripts to sex and age, as well as severity, indicate a confounding effect of these variables on the expression-severity relationship of these transcripts, thus warrant adjustment. Figure 1d,e illustrate the “biasograms” after an age by sex standardization. These figures show that standardization has no effect on severity correlated transcripts but as expected, transcripts that were originally correlated to age and sex become uncorrelated. A positive correlation of age to sex which signifies an age by sex interaction as a potential confounder on the gene expression-severity relationship was also observed (Fig. 1c) and eliminated after standardization (Fig. 1f).

### Global blood transcriptome profiles associate with RSV disease severity

**Figure 2:**
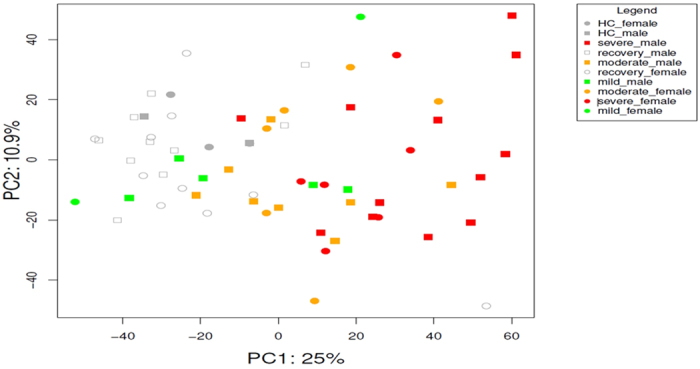
Global blood transcriptome profiling with principal component analysis: the first principal component (PC1) accounts for 25% of the variance in the dataset and associates with disease severity. This can be observed as a shift from healthy controls and recovery cases (left) through mild and moderate to severe cases (right), with considerable overlap.

Figure 2 illustrate a PCA on the whole transcriptome and the first principal component accounts for 25% of the variance in the transcriptomes and associates with disease severity. Transcriptome profiles of HC and recovery samples group together on the first principal component and are located opposite to profiles of severe infants. The distinct groups do not form discrete clusters in the PCA but gradually shift from mild through moderate to severe, with considerable overlap. This shows that the blood mRNA profiles substantially capture the severity of lower respiratory tract RSV infection.

### Number of differential gene expression relates to RSV disease severity

Table 2 presents results of differential gene expression analysis and reveals that the number of DE transcripts increases with disease severity. No DE transcript was identified between mild versus HC samples when applying a FDR of 5% and absolute fold change (FC) threshold of 2. However, 17 and 221 transcripts were DE between moderate and severe versus HC respectively. Interestingly, all transcripts that are DE in moderate class are also DE in severe class with larger FC. About 90% of these DE transcripts are up-regulated. Comparison of HC with recovery samples revealed a single down-regulated transcript while moderate versus mild yielded no DE transcript, severe versus mild or moderate yielded 178 and 49 DE transcripts respectively. Lastly, 95 transcripts were DE between severe versus combined mild/moderate samples.

### RSV induced blood transcriptome profiles reveal an inflammatory response

**Figure 3:**
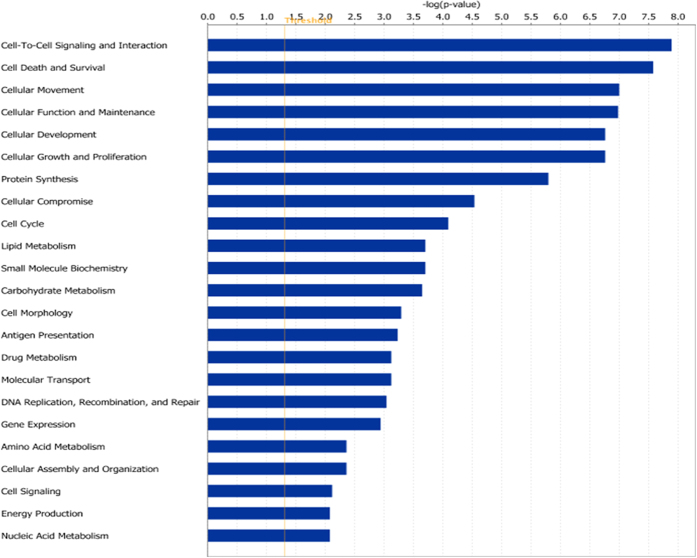
Ingenuity pathway analysis (IPA) Molecular and Cellular functions gene set analysis for severe vs healthy control contrast.

Figure 3 shows that multiple relevant categories of molecular and cellular functions are significantly enriched when comparing severe to HC samples. With “Cell-to-Cell Signaling and Interaction” top category, gene sets related to activation of several types of immune cells including lymphocytes, granulocytes and specifically neutrophils are most significantly enriched. In addition, gene sets that are involved in migration and tissue infiltration of these same activated cell types are most significantly enriched within the category “Cellular Movement” that ranks third on this figure. Finally, several high ranking molecular and cellular function categories and their underlying gene sets indicate the immune cells involved are strongly proliferating. A list of genes involved in each of these pathways is presented in supplementary Table S1. Taken together, blood transcriptome changes in RSV disease reveal a typical inflammatory response to a viral infection.

### Early blood transcriptome changes to predict a severe outcome of RSV infection

**Figure 4:**
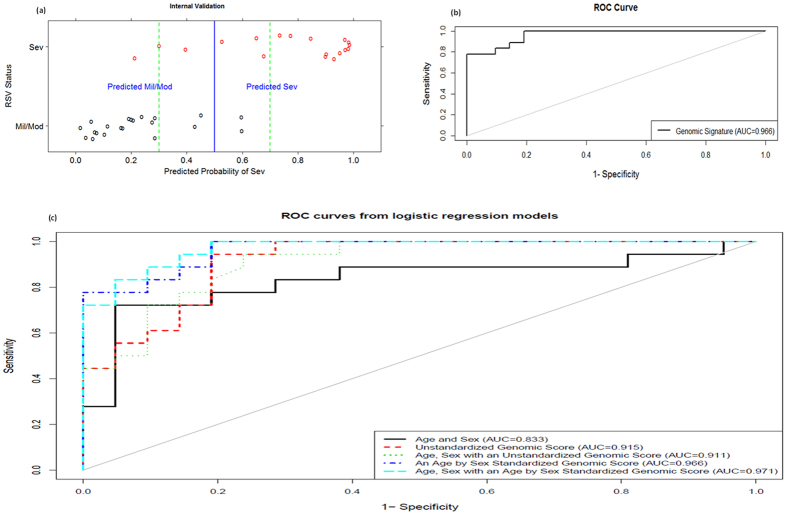
Internal validation of gene signature. (**a**) samples’ predicted probabilities of being severe. (**b**) shows the ROC curve and the AUC for predicted probabilities. The AUC value of approximately 1 indicates how accurate our signature performs on this internal validation set. (**c**) shows that a genomic model from the age by sex standardized data out performs that from the unstandardized data. In addition, there is a significant difference between a model with clinical parameters and that with a genomic score and just a slight improvement when both parameters are included in a model.

To construct a predictive model, we combined mild and moderate cases as a single group and three probabilistic classification functions were chosen based on supplementary Fig. S2 and results of Jong *et al*. and Kim and Simon^29^,^30^. Using these functions, classifiers were built and evaluated using LOOCV on the experimental data. SVM was chosen as the best calibrated and refined as shown on supplementary Fig. S3 and henceforth considered for all analyses. The LOOCV predicted probabilities from SVM were used to evaluate its performance and are plotted on Fig. 4a against the true RSV status as retrospectively determined. This figure shows that 5 samples out of 39 were misclassified at a 50% cutoff and when applying a proposed uncertainty band of 30–70% just one false negative is witnessed. Evaluation of the clinical characteristics of the single false negative patient as well as those patients with uncertain predictions (plotted within the proposed uncertainty band) did not reveal any recognizable pattern. The false negative patient had uniquely RSV and only a single patient plotted within the uncertainty band had RSV+ other virus(es). Figure 4b presents the corresponding ROC curve from the LOOCV predicted probabilities and AUC of 0.966 demonstrates the high discriminative power of our prognostic model.

### Genomic biomarkers outperform clinical parameters age and sex

Figure 4c presents the ROC curves from logistic models of clinical parameters and/or genomic scores. The genomic score model from the standardized data (AUC = 0.966) outperforms that from the unstandardized data (AUC = 0.915). In addition, there is a significant difference between a model with age and sex only (AUC = 0.833) and that with a standardized (AUC = 0.966) or unstandardized genomic score (AUC = 0.915). Whereas there is a slight improvement from clinical parameters and standardized genomic score model (AUC = 0.971), there is no improvement from the clinical parameters and unstandardized genomic score (AUC = 0.911), indicating that indeed standardization completely removed an age-by-sex effect on the gene expression data.

### An 84 gene expression signature predicts absence of disease progression

To extract the prognostic signature, we selected top transcripts maximizing the binomial log-likelihood function using LOOCV predicted probabilities as illustrated on supplementary Fig. S4. This figure depicts a 1-SE maximum of 95 transcripts corresponding to 84 unique genes, which are displayed on Table 3. Of the 95 transcripts constituting the prognostic signature, 81 (85.26%) were found to be significantly DE between severe and non-severe patients (FDR cutoff of 5%, supplementary Table S2). The inclusion of non-DE transcripts in the classification model is expected, since not only DE genes are instrumental in class discrimination as illustrated by the two-dimensional scenario in supplementary Fig. S5.

### Performance of the genomic signature retained on an independent dataset

**Figure 5:**
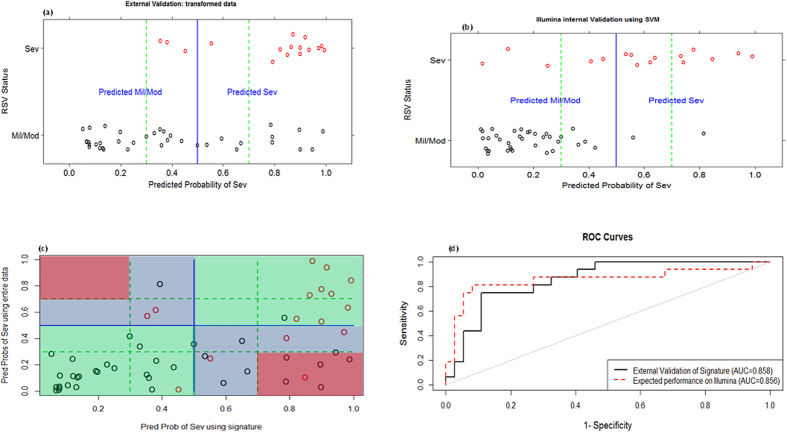
Predicted probabilities of being severe from the validation data against true RSV status using; our diagnostic signature (**a**) and LOOCV on unrestricted data (**b**). (**c**) illustrates the agreement of the predictions from both models, green regions are perfect agreement, blue are disagreements at a 50% cutoff and red are disagreements at a 30–70% uncertainty band. Finally, (**d**) presents the ROC curves and AUC from both models illustrating similar AUC values.

**Figure 6:**
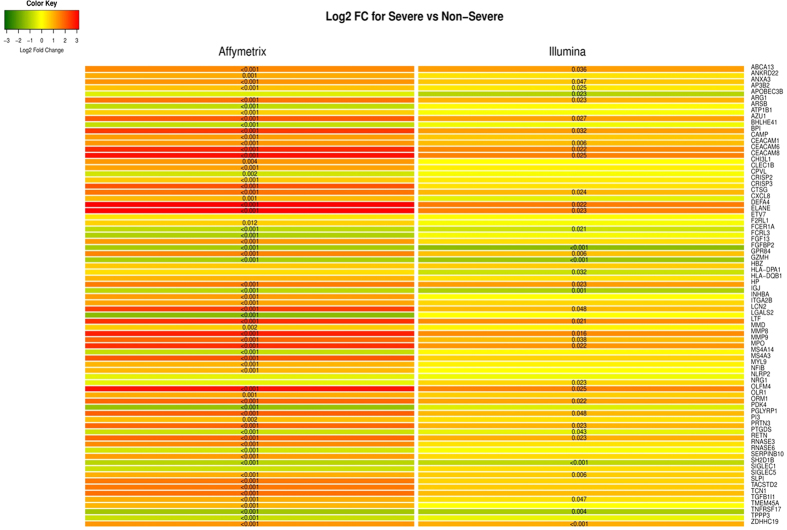
Log_2_ Fold change between Severe vs Non-Severe infants for 75 common genes in Affymetrix and Illumina datasets. Red represent up-regulation while green represents a down-regulation and the significant FDR adjusted p-values are placed in the cells. As one can clearly see, there is a huge overlap in the direction expressions across datasets. Where there are slight differences, these differences are not significant as shown by a non-significant p-value in at least one of the datasets.

For an independent validation, a subset of the Illumina RSV data of Meijas *et al*.^17^ was used. Since the experimental data and validation data were obtained using different platforms, we linked the data using gene symbols and applied cross-platform transformation (to render gene expression comparable across datasets) as shown on supplementary Fig. S6 and extensive described in the supplementary material. Figure 5a presents predicted probabilities of severe on the validation data using 75 of our 84 prognostic gene signature that were common in both experimental and validation datasets, while Fig. 5b presents the LOOCV predicted probabilities from SVM on the entire Illumina data. Both figures show that using the unrestricted data leads to more certain probabilities and slightly improves specificity compared to our signature. Nonetheless, Fig. 5c illustrates a large agreement between the predicted probabilities of the two models, while Fig. 5d clearly reveals that both models are alike as demonstrated by the AUCs of 0.858 and 0.856 for our signature and the unrestricted model respectively. To assess the concordance of the expression patterns of our signature on both datasets (Affymetrix and Illumina), we plotted the log_2_ fold changes of the common 75 genes as shown on Fig. 6. From this figure, one clearly sees that there is a huge concordance in the direction of expressions across datasets. Where there are slight differences, these differences are not significant as shown by a non-significant p-value in at least one of the datasets.

## Discussion

RSV infection in infants may cause life-threatening disease. No vaccine is yet available and triage of patients is challenging since RSV infections may rapidly progress to severe disease. No reliable prognostic model to predict which RSV patient will not progress to severe disease and could be safely send home is available either. Thus, clinical care is symptom-based and a significant proportion of RSV infected infants is hospitalized for observation purposes. We have provided an 84 gene signature that discriminates hospitalized infants with less severe RSV infection from those infants with severe RSV disease. The identified signature yielded a LOOCV AUC of 0.966 on the experimental data and was independently validated with an AUC of 0.858 and might serve as a basis to develop a prognostic test for clinical management of RSV disease.

In line with epidemiological observations^38^ and observations of Mejias *et al*.^17^, we showed the confounding effects of age and sex on gene expression-severity relationship for RSV disease. Studies in any RSV patient cohort with a naturally occurring “skewed” distribution of age and sex can be standardized for these parameters. By adjusting for an age-by-sex effect in our analyses, we obtained age-by-sex independent results which can be effectively applied to any patient(s). The high performance of our signature on the age and sex matched validation data signifies age-by-sex independence and robustness of this signature. Fewer co-infections were observed in severe patients (Table 1). A similar trend has been described previously^39^. In our cohort study we did not take into account co-infections since no consistent association between the occurrence or absence of co-infections with RSV disease severity have been reported^39^,^40^,^41^,^42^,^43^. Furthermore, we aimed at the identification of a gene signature in a natural “real-life” cohort of patients not stratified according to age or occurrence of co-infections.

We hypothesized that changes in blood cell type distribution and/or mRNA expression changes of the circulating cells collected from peripheral blood reflect local lung host response characteristics that associate with disease severity. PCA and DE analysis indeed revealed significant changes in the transcriptome profile of whole blood. Gene set analysis further shows that relevant processes are monitored including the activation, migration and tissue infiltration of lymphocytes, granulocytes and neutrophils. Individual DE genes in severe RSV disease revealed overexpression of the neutrophil associated genes MMP8 and MMP9, which have previously been related to severe RSV disease^44^. ARG1 and CHI3L1 that have been linked to alternatively activated macrophages in a mouse model for vaccine enhanced RSV disease^16^ were also found to be strongly up-regulated. This suggests that the collected blood transcriptome profiles indeed reflect local lung host response.

In our class prediction analysis, three functions were evaluated and the best was chosen. While it has been pointed by^45^,^46^,^47^,^48^ that selecting a minimal-error classifier leads to selection bias that should be corrected, the literature does not stipulate a selection bias when using calibration and refinement scores as evaluation measures. Nevertheless, we employed the nested cross-validation correction of selection bias^46^ in our model building procedure by splitting our experimental data into learning and test sets with an inner loop split on the learning set for parameter(s) optimization. Though found to contain high variance, we utilized leave-one-out cross-validation for the test set because it yields approximately an unbiased estimate of the true (expected) prediction error^49^ and because we were interested in the individual sample predicted probability of severe and not entirely on the expected predicted error. Nevertheless, where we were interested in the expected predicted error, as in the optimization of parameters, we utilized five-fold cross-validation as recommended by Breiman and Spector^50^. To validate the identified signature, an independent dataset generated on a different platform was used. Despite (i) the several sources of variability between our experimental data and the validation data that stem from - but not limited to - array platforms and different clinical cutoffs of RSV severity statuses, (ii) different time of profiling, 1–3 days after hospitalization and (iii) loss of information due to a reduction in signature because of no corresponding transcripts on Illumina platform and the aggregation of multiple transcripts to genes, our signature yielded an AUC of 0.858 that was comparable to accuracy (AUC of 0.856) when using the Illumina data (validation set) as experimental set. Cross-platform validation is rare due to lack of guidance on how this can be done reliably. We presented a cross-platform validation procedure.

The RSV patients enrolled in the study displayed varying disease severities but were all hospitalized thus representing a severe disease enriched subset of RSV infected infants. The patients enrolled however also represent a natural cohort of patients including a significant number of patients that eventually did not require extensive medical care and could have been discharged home. Since the blood samples were collected soon after hospital admission, the generated blood transcriptomes and the derived gene signature may serve as a basis for the development of a novel genomic tool to support clinical management of RSV disease including triage of patients presenting at the hospital provided that a rapid (real time) gene test can be developed. Larger transcriptome data sets are however required to construct predictive models that may also allow for discriminating mild from moderate and moderate from severe cases. Ultimately, one would like to extend the RSV biomarker program to earlier time point samples (e.g. obtained when visiting a general practitioner) and to samples collected from patients infected by other (respiratory) infectious agents or pathological conditions (comorbidities) in order to identify specific respiratory viral prognostic biomarkers. To this end a novel gene signature have to be developed using a much larger early blood sample cohort. The current results support the development of diagnostic tests for personalized medicine that not only provide information on the causative infectious agent, but also about the disease severity that may be expected.

## Additional Information

**How to cite this article**: Jong, V. L. *et al*. Transcriptome assists prognosis of disease severity in respiratory syncytial virus infected infants. *Sci. Rep*. **6**, 36603; doi: 10.1038/srep36603 (2016).

**Publisher’s note:** Springer Nature remains neutral with regard to jurisdictional claims in published maps and institutional affiliations.

## Supplementary Material

Supplementary Information

## Figures and Tables

**Table 1:** Patient characteristics (n represents the number of samples per group).

Parameters	Mild (n = 7)	Moderate (n = 14)	Severe (n = 18)
Age (days)	153 [84, 291]	185 [60, 333]	31 [17, 76]
Gestational age (weeks)	40 [29, 41]	40 [37, 41]	39 [37, 40]
Birth weight (kg)	3.5 [3.0, 4.2]	3.4 [3.1, 3.9]	3.3 [2.5, 4.0]
Symptomatic days	4 [2, 6]	4 [3, 6]	3 [2, 4]
Duration on O_2_ (days)	0	3 [2, 5]	8 [7, 11]
Ventilation	None	Supplemental	Mechanical
Length of stay (days)	4 [2, 6]	5 [3, 8]	11 [9, 13]
Breastfeeding	4 (57)	11 (79)	12 (67)
Male gender	5 (71)	10 (71)	12 (67)
RSV + other virus(es)	4 (57)	8 (57)	3 (17)

Data are presented as median and interquartile range (IQR) in square brackets [.] or number and percentage in brackets ().The median age of the healthy controls was 536 days (IQR [472, 602]).

**Table 2:** Number of differentially expressed transcripts for each contrast at FDR of 5% and absolute fold change cutoff of 2.

	Mil-HC	Mod-HC	Sev-HC	Mod-Mil	Sev-Mil	Sev-Mod	RC-HC	Sev-(Mil+Mod)/2
UP	0	15	194	0	164	42	0	82
Down	0	2	27	0	14	7	1	13
Total	0	17	221	0	178	49	1	95

Where Mil: Mild, Mod: Moderate, Sev: Severe, HC: Healthy controls (<2years) and RC: Recovery samples.

**Table 3:** Gene signature of 95 transcripts (probesets) corresponding to 84 genes.

N^o^	Affymetrix IDs	Gene Symbol	Illumina IDs
1	1553605_a_at	ABCA13	0pf.QRwb4sWsn7jQKQ
2	238439_at 239196_at	ANKRD22	oXed_glbkhVEMCDCTo xnW5awXa6P_EbICH0s HIf_t7qU.BejlFP86o
3	209369_at	ANXA3	orojh4FCCMN1ArUY6k
4	205678_at	AP3B2	9SneFS4fUDnuF7_nVM
5	206632_s_at	APOBEC3B	9Ff619hplIXH7nOK6I
6	206177_s_at	ARG1	0lVMTIuod_g0ohAnFE
7	232197_x_at	ARSB	3IDh9IVCveAqVdCl2o cMniVI7195NdYNUqeo BJIKm4IFqJ01E3eHp4 0u0lM7OqfU.quOIC94
8	201242_s_at	ATP1B1	unu3iN6N5U0f6cuEqc 9pU2OJP.BMdTLf9e64 fvOfs_Dnt_E3fl_11o
9	214575_s_at	AZU1	QBuHuFVihS7ZVBBu5E
10	221530_s_at	BHLHE41	EUN35CXUp3p.txedvU
11	205557_at	BPI	EqRaeX9VKg13UjfBQg
12	210244_at	CAMP	rItcu7lcV6dKfep3iA
13	209498_at 211889_x_at	CEACAM1	KpCYUm43RIkQFdLdQU fr.cFeEaHuTH9XTU54 xKXCKf3Uoo8n6L3cx0
14	203757_s_at 211657_at	CEACAM6	9criZPiUuucDnEx1B4
15	206676_at	CEACAM8	ubtBVA.8bOI1.LUVDo
16	209395_at 209396_s_at	CHI3L1	Zn_tzf4mVHIVHodF1U
17	208168_s_at	CHIT1	NA
18	220496_at	CLEC1B	uSC2Dit6C6ijp2vN0g
19	208146_s_at	CPVL	cdfjqToegKNUoMQgpI ZeEuC6evE1pCauH1NI
20	210262_at	CRISP2	ukOTfdVSNNEgNH9KQ4
21	207802_at	CRISP3	uqDXiAQ0SFE4hwohO4
22	205653_at	CTSG	0RKUnUS75SJfDAbUSI
23	202859_x_at	CXCL8	3Vy3nJSjUQtfvUe5fo EVLftPlJ7vrLnu_Dxo
24	207269_at	DEFA4	6530r9KdBCqTred_SM
25	206871_at	ELANE	NtF0nlRFRHdSTekQwE
26	224225_s_at	ETV7	9KCUpFjIkVJVFXsDoM
27	213506_at	F2RL1	BSHqBKlSg3u6xAXk64 ZAl9Iq.oUTd.oAy.kE
28	211734_s_at	FCER1A	WgUoCn0h94FRNcJQFU
29	231093_at	FCRL3	KV7kDSLO4uggquLXB4 ZUclXxUi6VEoBJeRT8 67unrLpPnjv_uzOezU
30	205110_s_at	FGF13	Hd7t51A7ISQ_3qyhSc Hvun37sCEoHuge477c TmCOYpTxe.jV7aAXpE
31	223836_at	FGFBP2	xmX900n31irhK4CFXE
32	223767_at	GPR84	ok0Fe53OSU4FlD1lCQ
33	210321_at	GZMH	rgmXcEkpOIcF9d6l4U
34	206647_at	HBZ	lteivVSVR8WbDmUMBQ
35	213537_at	HLA-DPA1	NFtdNMC3eb3pThValQ
36	203290_at	HLA-DQA1	NA
37	209480_at	HLA-DQB1	TfXanqXzTU0Si0gKnU
38	206697_s_at	HP	fpPOCkkS1WAIIRYIlc
39	215118_s_at	IGH	NA
40	212592_at	IGJ	HhecR84SAQucRJ7rVE
41	217148_x_at	IGLC1	NA
42	234764_x_at	IGLV1-44	NA
43	227140_at	INHBA	cp0iOCS4CISg_oqAqI
44	206494_s_at 206493_at 216956_s_at	ITGA2B	H3o0v7nktkZOvQbtdU
45	212531_at	LCN2	WUTbfV7VDYUuzYaeLk
46	208450_at	LGALS2	WT6GkkHqpCBBqCNF7k
47	222196_at	LOC389906	NA
48	238717_at	LOC441528	NA
49	1569110_x_at	LOC728613	NA
50	202018_s_at	LTF	cQO5fddUoFsfScd65c
51	244523_at	MMD	6p_X8jaueM_Xv1yw6k
52	207329_at 231688_at	MMP8	fgvcFUrFTg7ifFKt.k BelT8r_h3KIiQoopKI
53	203936_s_at	MMP9	fn3Hpm4vVouL4FK6FA
54	203949_at 203948_s_at	MPO	KXq9Q16d7p_cnO4×4k
55	229510_at	MS4A14	QqQF0qheQ0ghuCkj.Q 0JYKEkJ0rogLlAHvOU
56	210254_at 1554892_a_at	MS4A3	lKdKj.P0uB56B0ROV4 Qen_rt.er1_QxUxR9Q B3q9A94B7R88Xs4E6k
57	201058_s_at	MYL9	Tbs1URA5CdFCtV3S1U 3CBVEhgxeipOOJilWo
58	209290_s_at	NFIB	BNHPXrzdFBgpyimm0g QUlzHnJJ50×5LQ8egk
59	221690_s_at	NLRP2	EFIl20d6F6tINX6rcs
60	206343_s_at	NRG1	ruNF5QODCEECIJIKkI EX0VFaBXiuIGVC0kHQ rcTUUeoQlN_wDuaIKI 9QpESNQOTkjUnNJB00 uX15cu4f_VUIuXoST0 ZZlmuuNioCuRWSaaSQ
61	212768_s_at	OLFM4	rgjyBSHxwUPUVVFBVc c70LXLcyj6S.A5.HVU
62	210004_at	OLR1	9V0C7RDnXXjh11UgBQ
63	205040_at	ORM1	fG6ilCJP2dH5410qEU
64	227474_at	PAX8-AS1	NA
65	225207_at	PDK4	ESC1yEuE.fDrqLUnTk
66	207384_at	PGLYRP1	3mxHd1KQUncUXTUg_k
67	41469_at 203691_at	PI3	NeHlSg0ILnuCnfmo6U
68	207341_at	PRTN3	EX243qjuUVl.1eH30Y
69	211748_x_at 212187_x_at	PTGDS	HXlUBYwuThoEModVKk
70	220570_at	RETN	Qz8qQwkk_l6LlIm0XU
71	206851_at	RNASE3	9aLI9auX8dR7hdMDUo
72	213566_at	RNASE6	Te4VV0giY1VcQvr17E
73	214539_at	SERPINB10	f0LQV4ks6o4uOX0lAk
74	1553177_at	SH2D1B	0TlE1O9_rTf.5×9Oi0
75	219519_s_at	SIGLEC1	iiFGSdPjXjklE7iF0E
76	220000_at	SIGLEC5	Q13VUdxtx69ahqiNUw oJC4o.lSL0tenoolEk
77	203021_at	SLPI	No174RVAVBCigl6guU
78	202286_s_at	TACSTD2	WihIT5WgHS3Pz30n5U
79	205513_at	TCN1	i4uimBR4lCiesvG_1k
80	209651_at	TGFB1I1	EJXHlJXn14J32nhJWc iJVfHqoq1_Q9SYNopQ
81	219410_at	TMEM45A	6qievf0j6P7Xs1VS6I r_iBJ6cKOHcLse.k.U
82	206641_at	TNFRSF17	iWCth3hT5.UdUnOigo
83	218876_at	TPPP3	ENZUufqUJFe6TUJ1Xo QLe5eyXThVBUlUpOnA
84	231122_x_at	ZDHHC19	69eJXi6CX97l_V.lR0
