# Supplementary material for: Transcriptome assists prognosis of disease severity in respiratory syncytial virus infected infants
Source: Sci Rep. 2016 Nov 11;6:36603. doi: 10.1038/srep36603 (PMC5105123; doi:10.1038/srep36603)
Supplement: Supplementary Information [file srep36603-s1.pdf]

Transcriptome assists prognosis of disease severity in respiratory syncytial virus infected infants

Victor L. Jong, Inge M.L. Ahout, Henk-Jan van den Ham, Jop Jans, Fatiha Zaaraoui-Boutahar, Aldert Zomer, Elles Simonetti, Maarten A. Bijl, H. Kim Brand, Wilfred F.J. van IJcken, Marien I. de Jonge, Pieter L. Fraaij, Ronald de Groot, Albert D.M.E. Osterhaus, Marinus J. Eijkemans, Gerben Ferwerda, and Arno C. Andeweg

Online Data Supplement

Material and Methods

Study design

Nasopharyngeal wash (using Cheiron Dynamic II apparatus) and blood samples were prospectively obtained from patients less than 2 years of age with a bronchiolitis. Patient enrolment occurred 7 days a week and samples were taken within 24 hours after first contact with the hospital. Only patients with an RSV infection, as determined by PCR retrospectively, were included in the study. Exclusion criteria were: immunodeficiency, systemic steroid treatment in the previous 2 weeks, blood transfusion, congenital heart and chronic lung disease. Patients were followed until recovery and were retrospectively classified as: mild for children without hypoxia, moderate for patients requiring supplemental oxygen (oxygen saturations <90%, ≥10 minutes) and severe for children requiring mechanical ventilation due to apnea, exhaustion and/or respiratory failure. Recovery samples were obtained after 4-6 weeks, during home visits. Blood samples were obtained from healthy patients without underlying diseases or medication subjected to elective surgery.

Sample processing and blood transcriptome profiling

Multiplex RT-PCR was performed to test the nasopharyngeal washes on 15 different viral pathogens, as previously described (E1). Blood was collected in Tempus tubes and stored at -80°C. Total RNA was isolated from the blood using Tempus Spin RNA isolation kit (Applied Biosystems, Bleiswijk, The Netherlands). Globin mRNA was removed from total RNA preparations using the Globinclear kit (Life Technologies). RNA concentrations and OD 260/280 ratios were measured with the NanoDrop ND-1000 UV-VIS spectrophotometer (NanoDrop Technologies, Wilmington, USA). Assessment of RNA quality and purity was performed with the RNA 6000 Nano assay on the Agilent 2100 Bioanalyzer (Agilent Technologies, Palo Alto, CA, USA). RNA (200ng) was labelled using the MessageAmp Premier RNA Amplication kit (Applied Biosystems) and hybridized to Human Genome U133 plus 2 gene chips (Affymetrix), according to the manufacturer's recommendations. Image analysis was performed using GeneChip Operating Software (Affymetrix). Microarray Suite version 5.0 software (Affymetrix) was used to generate .dat and .cel files.

Differential expression analysis

For $j = 1 \dots n$ samples and $i = 1, \dots, p$ transcripts (probesets) the linear model for each probeset was as follows:

$$Y_{ij} = \boldsymbol{\beta}_i \boldsymbol{Status_j} + \alpha_{i1} Sex_j + \alpha_{i2} Age_j + \alpha_{i3} Sex_j * Age_j + \varepsilon_{ij} \quad (1)$$

Where Y_i is a vector of the expression values of probeset i, Status is an indication matrix of the samples by RSV categories and β_i is a vector of coefficients of the RSV categories for

probeset i. The DE analysis was then performed by comparing the contrasts of ***Status*** for each probeset.

Identification and evaluation of prognostic biomarkers

Given that we are interested in genomic prognostic biomarker(s), we retained a sex by age standardized dataset by fitting the linear models in equation (1) above using *limma* (E2) and from those models the sex by age standardized expression set was:

$$\hat{Y}_{ij} = \boldsymbol{\beta}_i \boldsymbol{Status_j} + \varepsilon_{ij} \quad (2)$$

As class labels, we combined the mild and moderate groups as one class (class 0) with focus on predicting severe cases (class 1) from others. For clinical application, a prediction of the probability to progress to severe disease is of primary interest than direct classification (E3). Therefore, using results of (E4, E5) and the observed correlation distribution shown on supplementary Fig. S2 three classification functions were chosen. These classification functions were support vector machines (SVM) with a linear kernel (E6,), shrunken centroids discriminant analysis (SCDA) also known as prediction analysis of microarray (PAM) (E7) and random forest (RF) (E8).

For each classification function, a prediction model was built and evaluated using leave one out cross validation with an inner loop of 5-fold cross validation for parameter(s) optimization as shown on Fig. S6 step 1, optimizing the parameters by maximizing the binomial log-likelihood function. We evaluated the prediction models using calibration score (CS) and refinement score (RS) (E5) which is a decomposition of the Brier score. The calibration score expresses on one hand, how well the predicted probabilities agree with the true chances of patients and it is equal to zero in case of perfect agreement. On the other hand, the refinement score expresses how uncertain the predicted probabilities are; that is how close the predicted probabilities are to 0.5. The closer the predicted probabilities are to 0.5, the higher the uncertainty and the poorer the model. A good class prediction model has a CS and RS of zero. The best calibrated and refined function amongst the three classification functions (Fig. S3) was chosen and its performance evaluated using the area under the receiver operating characteristic (ROC) curve (AUC) as shown on Fig. S6 step 2. Whereas a ROC curve is a plot of the true positive rate against the false positive rate for the different possible cut-points of the prediction model, AUC measures the accuracy of the model. An AUC of 1 represents a perfect model and an AUC of 0.5 represents a worthless model.

For the chosen classification function, the model was built on the entire dataset with a 5-fold cross validation for parameter(s) optimization based on maximizing the binomial log-likelihood function. The list of transcripts from the optimal parameter(s) was retained (Fig. S6, step 3) as a gene signature.

Logistic regression models

Let p_i be the leave one out cross-validated predicted probability of sample i to progress to severe state by a genomic signature, then the genomic score is given as: $GS_i = log\left(\frac{p_i}{1-p_i}\right)$. The general logistic regression model is then written as:

$$log\left(\frac{\pi(x_i)}{1-\pi(x_i)}\right) = \beta_0 + \boldsymbol{\beta_1 P}_i \qquad (3)$$

where $\pi(x_i)$ is the probability of sample i to progress to severe state, $\boldsymbol{P}$ a vector or matrix of predictive parameters (i.e. Genomic score, Age, and/or Sex) and $\boldsymbol{\beta_1}$ is a vector of parameter(s) estimate(s). Let y_i be the predicted value of sample i from equation (3), the probability of that sample to progress to severe state is computed from its predicted value using the inverse logistic function as: $\pi(x_i) = \frac{e^{y_i}}{1-e^{y_i}}$. With these predicted probabilities, the AUCs were computed.

Validation of biomarkers

For an independent (external) validation, a subset of the Illumina RSV data set of (E9) was used. Since the experimental data and validation data are from different platforms, we opted to link the data using gene symbols. To achieve this, the signature transcripts were annotated to gene symbols and unannotated transcripts were eliminated if any (Fig. S6, step 4). The Illumina data was also annotated to gene symbols (Fig. S6, step 5) and the common genes between our annotated gene signature and the annotated Illumina data were extracted using common gene symbols as shown in step 6 of Fig. S6 and were referred to as the surrogate signature (step 7). The final expression set of the surrogate signature was computed by assigning the median expression value of a gene with multiple transcripts to the corresponding gene (step 8) and was referred to as unique surrogate signature. The final model was then built with the unique surrogate signature expression set from the Affymetrix data (step 9) and validated with the unique surrogate signature expression data from Illumina (step 12).

To be able to validate with the Illumina data, all the beads corresponding to the genes in the surrogate signature were retrieved (step 10) and multiple beads per gene were combined by calculating their median expression values (step 11). Since the expression values of a single gene might be measured on different scales across Illumina and Affymetrix platforms, we rescaled the Illumina data of the gene signature to same scale as Affymetrix data (step 12). Suppose for each gene *i* the expression scale in Affymetrix is in the interval [a_i, b_i] and [c_i, d_i] in Illumina. To transform an expression value x_{ij} of gene *i* in sample *j* from Illumina to Affymetrix scale, we use the following function:

$$f(x_{ij}) = a_i + \frac{(b_i - a_i) \times (x_{ij} - c_i)}{(d_i - c_i)} \qquad d_i \neq c_i \qquad (4)$$

The scaled Illumina data was then predicted using our prognostic model (step 12) and the validating ability of the model was evaluated (steps 13 and 14) of the same Fig. S6. Worth to mention is the fact

that this transformation does not affect the expression values of genes with same scale on Affymetrix and Illumina platforms and since there is no gene with a constant expression value, the function stays defined.

For a confirmatory analysis of our validation performance, using the chosen classification function we built class prediction model on the entire Illumina data using LOOCV as shown on step 1 of Fig. S6. We then compared our validation performance to the performance that can be achieved on this data with the chosen function.

Figures and Tables

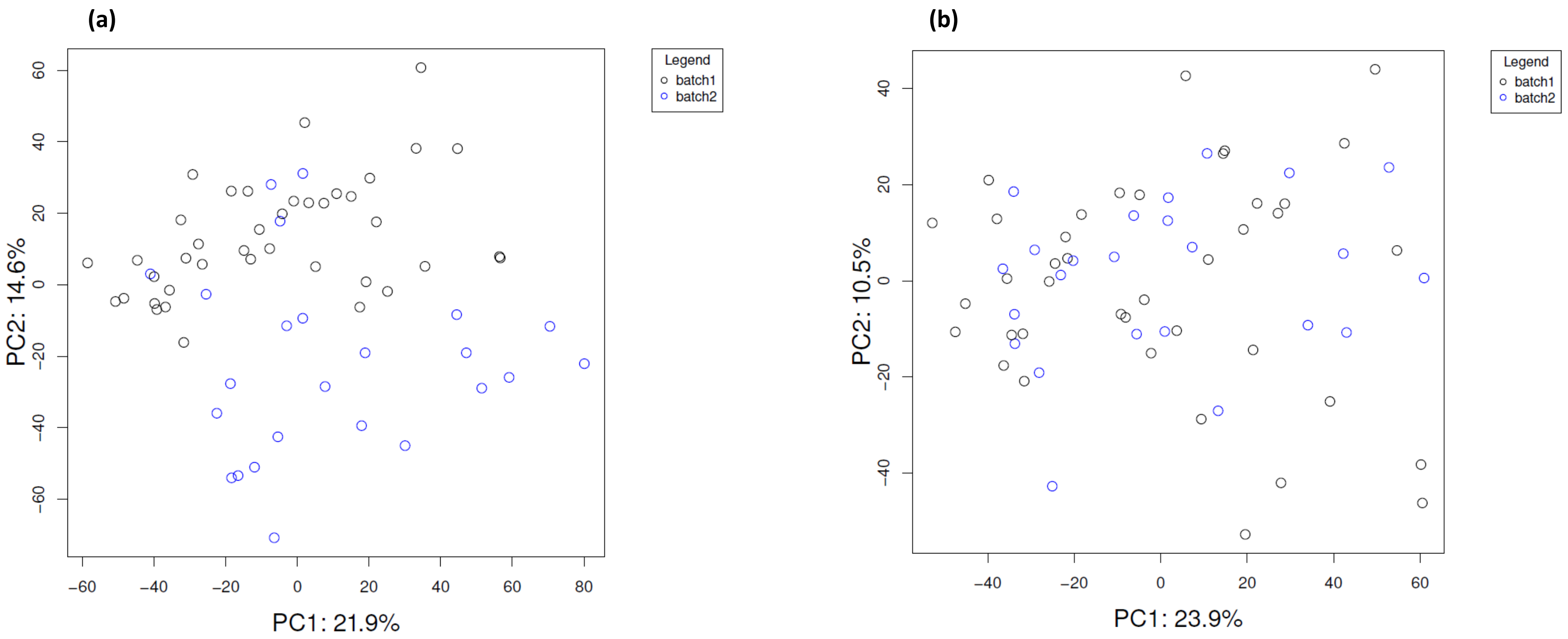

Figure S1: *Principal component analysis (PCA) of all probesets before* ***(a)*** *and after* ***(b)*** *batch adjustment. It can clearly be seen that before batch adjustment samples from both batches cluster together on PC2 while such clusters disappear after batch adjustment.*

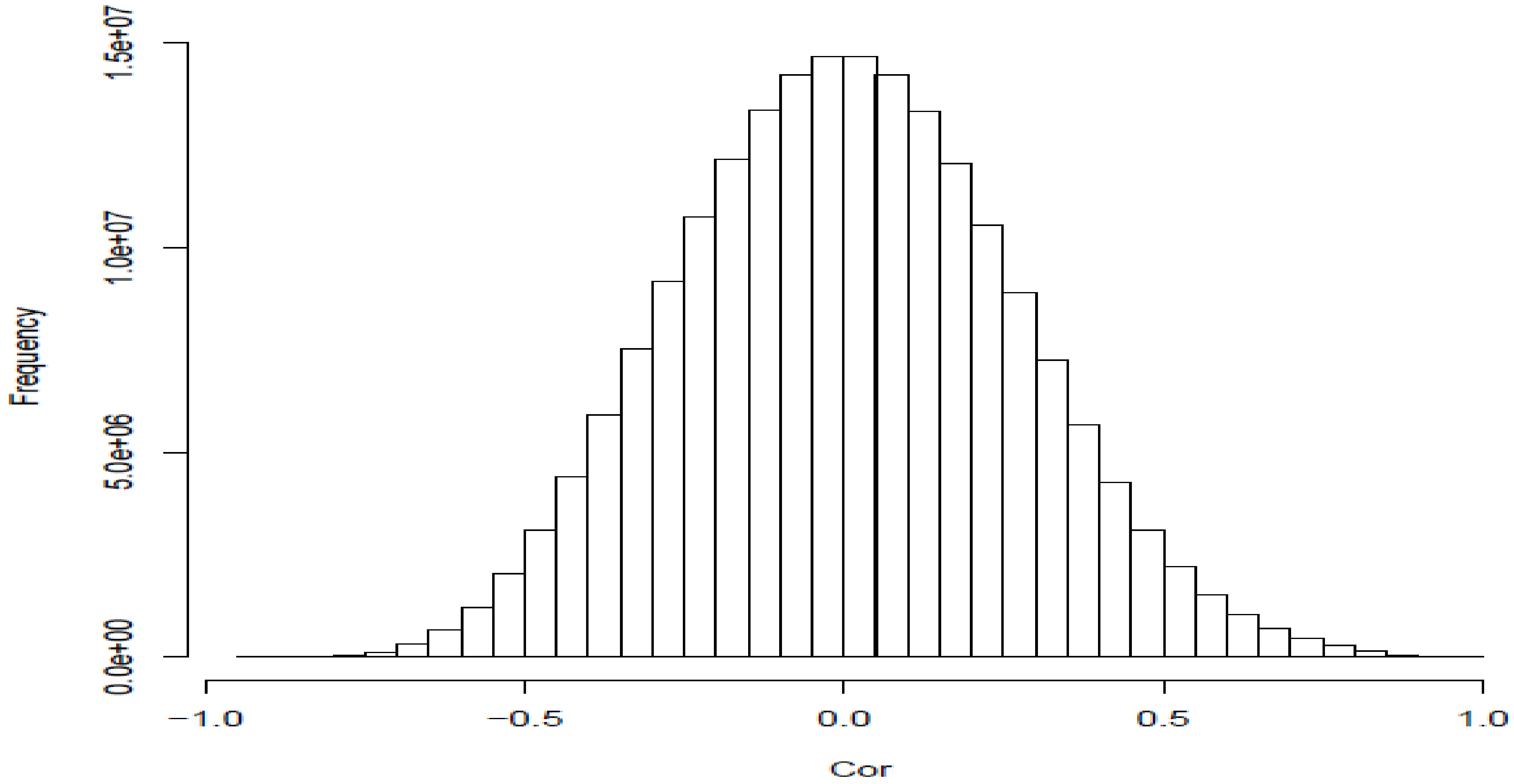

Figure S2: *Distribution of transcripts' pair-wise correlation values (cor) for the RSV experimental data. This figure clearly shows a low proportion of highly correlated genes. As such, the data might be classified as lowly correlated data.*

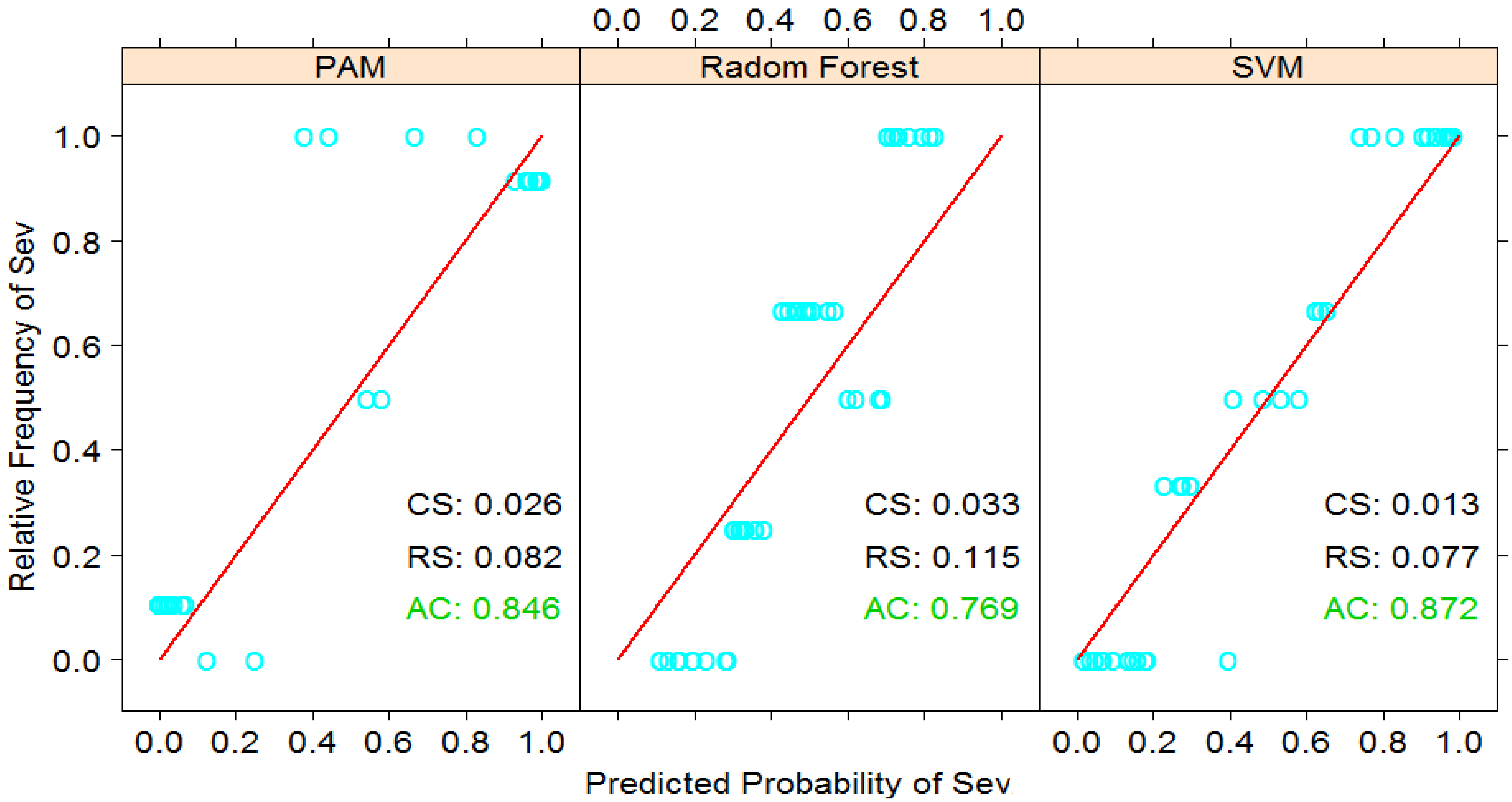

Figure S3: *A plot of the predicted probabilities and the relative frequencies of severe (Sev). Relative frequencies are computed over 10 bins using binning on the probability interval [0, 1]. Calibration score (CS), refinement scores (RS) and accuracies (AC) are presented for each classification function. Of the three classification functions, SVM outperforms PAM and RF as the most calibrated and refined.*

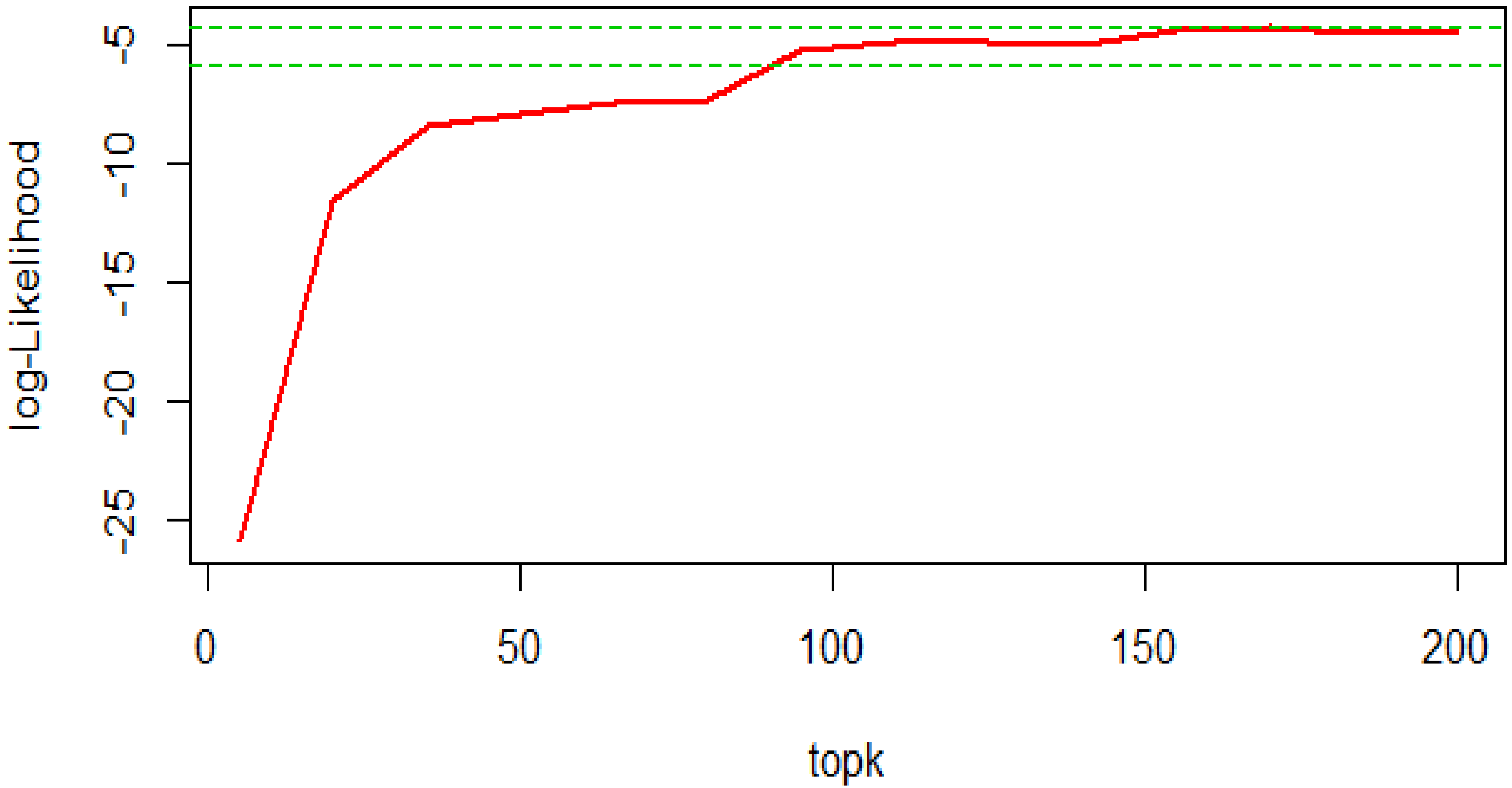

Figure S4: *To extract the prognostic signature, the same SVM with a linear kernel was built with the entire experimental data and a 5-fold cross-validation for the optimization of the cost parameter. Using the coefficients of the support vectors, the weights of the transcripts were computed and the transcripts were ranked by their absolute weights. The same 5-fold cross-validation used to optimized parameters was used to determine which top number of transcripts yielded a one standard error (1-SE) maximum binomial log-likelihood. The green dotted lines represent the MAX and MAX-1SE of the log-likelihoods. The first value to fall within the band is considered the 1-SE maximum and corresponds to 95 transcripts.*

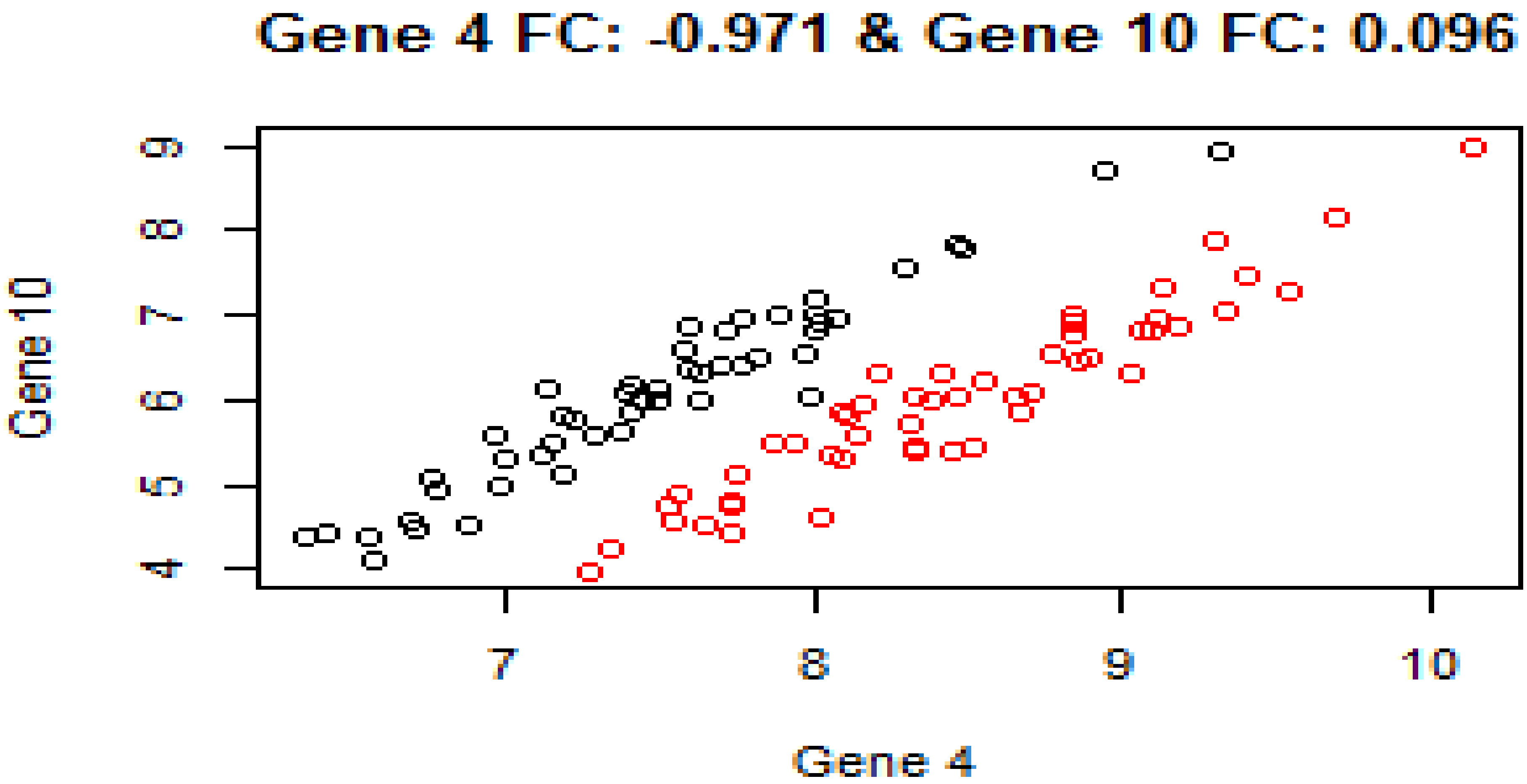

Figure S5: *A simulated 2-dimensional plot of two genes named Gene 4 and Gene 10 which individually do not separate the groups (black from red). Gene 4 can be considered differentially expressed (DE) with an absolute fold change (FC) of 0.971 but Gene 10 is definitely not DE with a small FC of 0.096 but a combination of the two separates the group almost perfectly. This illustrates the use of non-differentially expressed genes in class discrimination.*

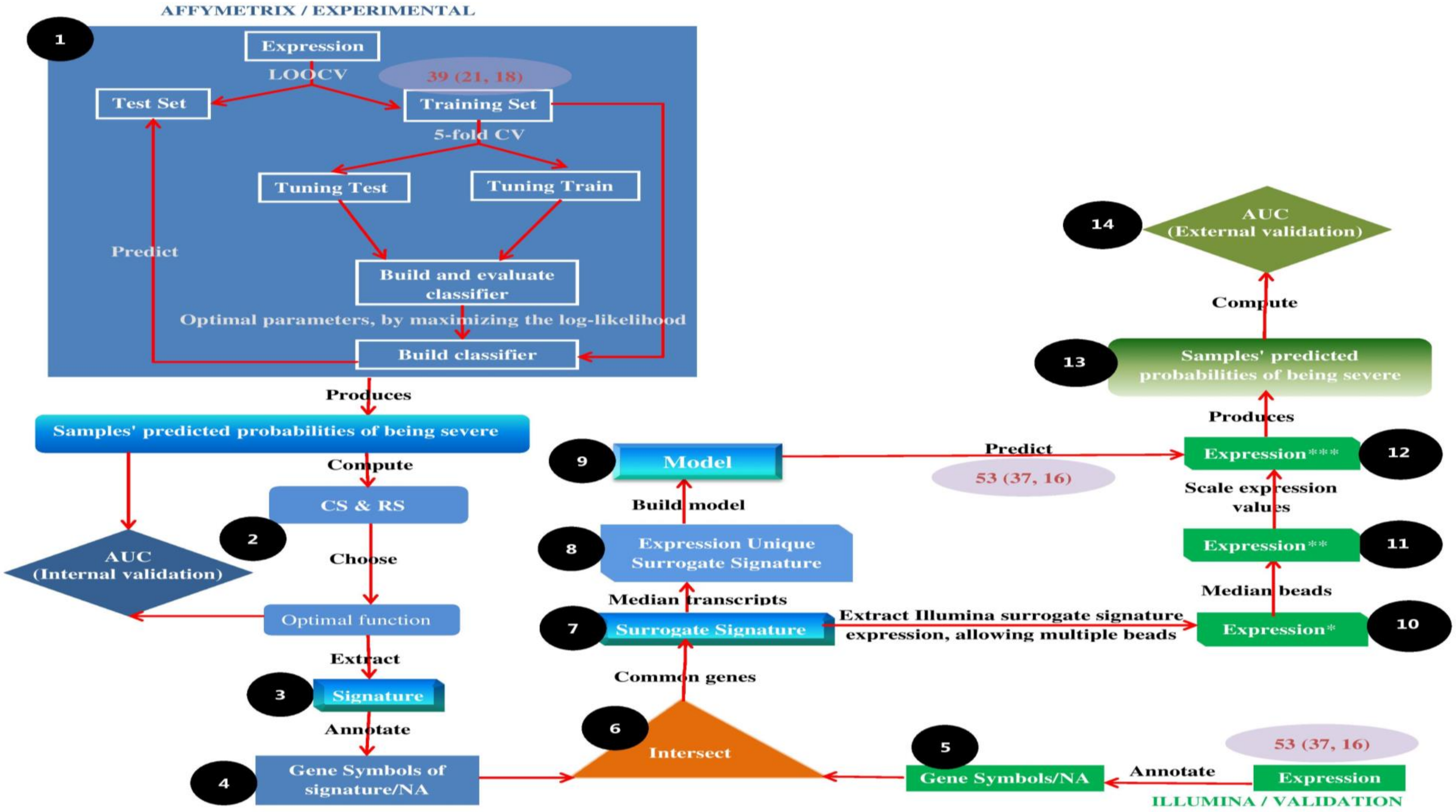

Figure S6: *Algorithm for building, evaluating and validating the class prediction model. Red numbers in ovals represent total sample sizes, outside brackets and per class sample size in brackets. Blue indicate analysis with Affymetrix data while Green indicate analysis with Illumina data. Where *: surrogate signature, **: unique surrogate and ***: scaled unique surrogate signature of Illumina expression values.*

Table S1: *List of genes involved in each and every pathway identified by IPA.*

Categories	# Genes	Genes
**Cell-To-Cell Signaling and Interaction**	52	AHNAK, BCL3, BID, BPI, CARD11, CD1C, CD1E, CD58, CD59, CD63, CEACAM1, CEACAM3, CLEC5A, DOK3, F5, FCAR, FCGR1A, FKBP1A, FPR1, GADD45A, GNAI2, HLA-DMB, HLA-DQB1, HLA-DRA, HLA-DRB1, IGHM, IL1RN, IRF8, KIR3DL1, KLRB1, LAX1, LTB4R, LY96, LYST, MAPK14, MMP9, NFATC2, NFATC3, NFKBIZ, PIK3CB, PLSCR1, PVRL2, RAB27A, RETN, RORA, S100A12, S100A8, SAMSN1, SBNO2, SEMA4A, SLPI, ST6GAL1, STAT3, STAT5B, TNFRSF18
**Cell Death and Survival**	138	AATK, ABCB1, ACVR1B, ADAM12, ADM, ARG1, B4GALT5, BACH2, BCL3, BHLHE41, BID, BMX, BPI, BTG1, C11orf82, CA4, CAMK2D, CD59, CEACAM1, CFLAR, CLEC5A, CMIP, CREBL2, CTSD, DNASE1L3, DRAM1, E2F1, E2F3, EEF1D, EFHC1, EHD1, EIF3F, EIF4B, ETV6, FCER1A, FCGR1A, FKBP1A, FLNB, FURIN, G0S2, GADD45A, GLTSCR2, GNAI2, GNB2L1, GRB10, HIST1H1C, HRK, HSPA8, IFI16, IGHM, IL10RA, IL1RN, IL23R, IRAK3, IRF8, ITGA7, KDM6B, KIDINS220, KIF1B, KIR3DL1, KLF7, KRAS, LIMK2, LMO2, MAPK14, MEF2C, MGP, MITF, MLLT11, MMP8, MMP9, MTF1, MYBL1, NAIP, NAPA, NBN, NCAM1, NCOA3, NDST1, NFATC1, NFATC2, NFKBIZ, PAFAH2, PALLD, PDLIM7, PDXK, PGLYRP1, PIK3CB, PIM3, PKM, PLSCR1, PPIF, PPP3CC, PRMT2, PTGDS, PVRL2, RAB27A, RASSF6, RBBP4, RHOG, RPL10, S100A8, SAT1, SEMA4A, SH3GLB1, SIGLEC5, SLC25A6, SLC2A3, SLC40A1, SLPI, SMAD5, SOCS3, SOD2, SORT1, SOX5, SPIB, SRPK1, ST6GAL1, STAT3, STAT5B, TCF3, TCF4, TCF7L2, TDRD9, TFPI, TIAM1, TLR6, TNFRSF18, TOP2B, TPD52, TRIM2, UBE2V1, UGCG, XCL1, YY1, ZC3H12A, ZFP36L2, ZMAT3
**Cellular Movement**	60	ADM, ARG1, BACH2, BID, CD58, CD63, CEACAM1, CLEC5A, DOCK8, ETV6, F11R, FCAR, FCER1A, FLOT1, FPR1, FYB, GBA, GNAI2, GNB2L1, GPR183, HP, IL10RA, IL17RA, IL1RN, KDM6B, KRAS, LTB4R, LY96, LYST, MAPK14, MLLT11, MMP8, MMP9, NDST1, NFATC1, NFATC2, NFKBIZ, PGLYRP1, PIK3CB, PRMT2, PTGDS, RAB27A, RETN, RPL13A, S100A12, S100A8, SEMA4A, SERPINB1, SLPI, SOCS3, SOD2, STAT3, STAT5B, TET2, TFPI, TIAM1, TNFRSF18, TRIO, XCL1, ZC3H12A
**Cellular Function and Maintenance**	43	ABCB1, AHNAK, BACH2, BCL3, BST1, CARD11, CEACAM1, DOCK8, F11R, FCER1A, FCGR1A, FLOT1, FYB, GNAI2, GPR183, GPR84, HLA-DQB1, IGHM, IL10RA, IL23R, IRF8, KLRB1, LAX1, LTB4R, LY96, MMP9, NFATC1, NFATC2, NFKBIZ, RHOG, SAMSN1, SEMA4A, SERPINB1, SH2D1B, SLPI, SPPL2A, ST6GAL1, STAT3, STAT5B, TAPBP, TCF4, TLR6, TNFRSF18
**Cellular Development**	51	AHNAK, ARG1, B3GNT5, BCL3, CARD11, CD58, CD59, CEACAM1, CFLAR, CTSD, DOCK8, E2F1, FKBP1A, FYB, GADD45A, GNAI2, GPR183, HLA-DQB1, HLA-DRB1, HLA-DRB3, HSPA8, IGHM, IL10RA, IL1RN, IRF8, KLRB1, KRAS, LY96, MAPK14, MEF2C, MMP9, MYBL1, NFATC1, NFATC2, NFATC3, PCYT1A, PIK3CB, PIM3, PLEKHA1, RHOG, SAMSN1, SLPI, SOCS3, SOX5, SPIB, ST6GAL1, STAT3, STAT5B, TCF3, TNFRSF18, XCL1
**Cellular Growth and Proliferation**	50	AHNAK, ARG1, B3GNT5, BCL3, CARD11, CD58, CD59, CEACAM1, CFLAR, CTSD, DOCK8, E2F1, FKBP1A, FYB, GADD45A, GNAI2, GPR183, HLA-DQB1, HLA-DRB1, HLA-DRB3, HSPA8, IGHM, IL1RN, IRF8, KLRB1, KRAS, LY96, MAPK14, MEF2C, MMP9, MYBL1, NFATC1, NFATC2, NFATC3, PCYT1A, PIK3CB, PIM3, PLEKHA1, RHOG, SAMSN1, SLPI, SOCS3, SOX5, SPIB, ST6GAL1, STAT3, STAT5B, TCF3, TNFRSF18, XCL1
**Protein Synthesis**	21	B3GNT5, BACH2, BCL3, BST1, CARD11, CLEC4D, DOCK8, FCGR1A, GADD45A, GNAI2, HLA-DQB1, IGHM, LAX1, MYBL1, NFATC1, NFATC2, RFTN1, SAMSN1, SPIB, SPPL2A, ZC3H12A
**Cellular Compromise**	15	CARD11, CD59, CD96, CEACAM1, CFLAR, DOCK8, FCGR1A, HLA-DRB1, KIR3DL1, KLRB1, NFATC1, NFATC2,

		RAB27A, STAT5B, XCL1
**Cell Cycle**	3	E2F1, PPIF, STAT3
**Lipid Metabolism**	3	ABCB1, GBA, UGCG
**Small Molecule Biochemistry**	3	ABCB1, GBA, UGCG
**Carbohydrate Metabolism**	15	B3GNT8, DYRK2, GRB10, GYG1, HS3ST3B1, IL1RN, KRAS, NDST1, PCYT1A, PPP1R3D, RETN, SOCS3, STAT3, TCF7L2, TNFAIP6
**Cell Morphology**	15	ABCB1, B3GNT5, BACH2, BCL3, CARD11, E2F1, HLA-DQB1, IGHM, IL1RN, IRF8, LYST, NFKBIZ, SPIB, STAT5B, TCF3
**Antigen Presentation**	9	CD1C, CD1E, CLEC4D, CTSD, FCGR1A, IGHM, MAPK14, SEMA4A, TAPBP
**Drug Metabolism**	2	ABCB1, UGCG
**Molecular transport**	2	ABCB1, UGCG
**DNA Replication, Recombination, and Repair**	13	BCL3, C11orf82, DYRK2, GADD45A, IFI16, NABP1, NBN, NFATC2, SESN1, SP100, USP28, YY1, ZBTB4
**Gene Expression**	33	ACVR1B, ADAM12, BCL3, CRIM1, E2F1, E2F3, ETV6, FGF13, GADD45A, IFI16, IRF8, LMO2, MAPK14, MEF2C, MITF, MMP9, MYBL1, NCOA3, NFATC1, NFATC2, NFE2, PRMT2, RORA, SOCS3, SPIB, STAT3, STAT5B, TCF3, TCF4, TCF7L2, TGIF1, YY1, ZBTB10
**Amino Acid Metabolism, Small Molecule Biochemistry**	2	ARG1, GLS
**Cellular Assembly and Organization**	2	NAPA, STXBP3
**Cell Signaling**	21	BCL3, CARD11, EEF1D, FGF13, FKBP1A, GADD45A, GNAI2, GRB2, IGHM, IL23R, MEF2C, NDST1, RORA, S100A12, SECTM1, SOCS3, STAT3, STAT5B, TLE1, TLR6, UBE2V1
**Energy Production**	7	G0S2, KRAS, PID1, PKM, PPIF, SOCS3, STAT3
**Nucleic Acid Metabolism**	7	G0S2, KRAS, PID1, PKM, PPIF, SOCS3, STAT3

Table S2: *Differentially expressed transcripts for the three contrasts and those that were included in the prognostic signature (shaded in green)*

N°	Probeset	Mod - Mil		Sev -Mil		Sev - Mod		Sev-(Mil & Mod)		Pred. Sign.
		LFC	Adj.P	LFC	Adj.P	LFC	Adj.P	LFC	Adj.P	
1	206871_at	0.808	0.952	3.627	0.000	2.819	0.000	3.223	0.000	YES
2	203949_at	0.804	0.952	3.163	0.000	2.359	0.000	2.761	0.000	YES
3	211657_at	0.892	0.952	3.177	0.000	2.285	0.000	2.731	0.000	YES
4	207269_at	1.284	0.952	4.025	0.000	2.741	0.001	3.383	0.000	YES
5	205557_at	0.777	0.952	2.951	0.000	2.174	0.000	2.562	0.000	YES
6	203757_s_at	1.041	0.952	3.347	0.000	2.306	0.001	2.827	0.000	YES
7	203948_s_at	0.828	0.952	3.073	0.000	2.245	0.001	2.659	0.000	YES
8	231688_at	1.574	0.952	3.815	0.000	2.241	0.004	3.028	0.000	YES
9	207341_at	0.352	0.957	2.114	0.000	1.762	0.000	1.938	0.000	YES
10	207384_at	0.522	0.952	2.339	0.000	1.818	0.001	2.079	0.000	YES
11	212531_at	0.965	0.952	3.004	0.000	2.038	0.002	2.521	0.000	YES
12	207329_at	1.173	0.952	3.537	0.000	2.364	0.002	2.950	0.000	YES
13	206676_at	1.145	0.952	3.689	0.000	2.545	0.002	3.117	0.000	YES
14	227140_at	0.219	0.960	1.401	0.001	1.182	0.001	1.291	0.000	YES
15	210254_at	0.740	0.952	2.712	0.000	1.972	0.002	2.342	0.000	YES
16	214575_s_at	0.427	0.957	2.326	0.001	1.900	0.001	2.113	0.000	YES
17	220570_at	0.347	0.960	2.068	0.001	1.721	0.001	1.895	0.000	YES
18	203021_at	0.515	0.952	2.038	0.001	1.523	0.002	1.781	0.000	YES
19	202018_s_at	1.284	0.952	3.405	0.000	2.121	0.009	2.763	0.000	YES
20	206851_at	0.624	0.952	1.877	0.000	1.252	0.006	1.565	0.000	YES
21	205653_at	0.562	0.952	2.089	0.001	1.527	0.004	1.808	0.000	YES
22	235816_s_at	0.337	0.952	1.279	0.001	0.942	0.004	1.111	0.000	NO
23	205033_s_at	1.043	0.952	2.876	0.000	1.833	0.010	2.354	0.000	NO
24	203936_s_at	1.242	0.952	2.736	0.000	1.494	0.022	2.115	0.000	YES
25	212768_s_at	2.289	0.952	4.408	0.000	2.119	0.051	3.264	0.000	YES
26	225782_at	0.335	0.952	1.395	0.001	1.060	0.004	1.228	0.000	NO
27	231093_at	0.156	0.967	-0.893	0.014	-1.050	0.001	-0.972	0.000	YES
28	205513_at	0.985	0.952	2.428	0.001	1.443	0.020	1.936	0.000	YES
29	219890_at	0.906	0.952	1.817	0.000	0.911	0.054	1.364	0.000	NO
30	226188_at	0.279	0.952	1.097	0.002	0.818	0.008	0.958	0.000	NO
31	206208_at	0.525	0.952	1.205	0.001	0.680	0.031	0.942	0.000	NO
32	214539_at	0.353	0.953	1.479	0.003	1.126	0.008	1.303	0.000	YES
33	231122_x_at	0.381	0.953	1.588	0.003	1.207	0.008	1.398	0.000	YES
34	202286_s_at	-0.133	0.987	1.616	0.014	1.749	0.001	1.682	0.000	YES
35	213541_s_at	0.233	0.953	1.045	0.003	0.813	0.008	0.929	0.000	NO
36	209651_at	0.010	0.998	1.019	0.010	1.009	0.002	1.014	0.000	YES
37	223767_at	0.746	0.952	2.043	0.002	1.297	0.020	1.670	0.000	YES
38	206464_at	0.599	0.952	1.488	0.001	0.889	0.029	1.188	0.000	NO
39	207802_at	0.768	0.952	2.656	0.003	1.888	0.014	2.272	0.000	YES
40	200696_s_at	0.198	0.959	1.006	0.005	0.808	0.008	0.907	0.000	NO
41	209498_at	1.098	0.952	2.180	0.001	1.082	0.080	1.631	0.001	YES
42	225207_at	-0.231	0.967	-1.461	0.008	-1.231	0.008	-1.346	0.000	YES
43	1554892_a_at	0.711	0.952	2.403	0.004	1.692	0.017	2.048	0.000	YES
44	220496_at	0.239	0.962	1.322	0.007	1.082	0.009	1.202	0.000	YES
45	1553952_at	0.217	0.958	1.010	0.007	0.793	0.013	0.901	0.000	NO
46	1553605_a_at	0.355	0.959	1.704	0.007	1.349	0.013	1.527	0.000	YES
47	203725_at	0.297	0.952	1.056	0.005	0.760	0.018	0.908	0.000	NO
48	206440_at	0.545	0.952	1.229	0.003	0.684	0.065	0.956	0.001	NO
49	1553177_at	-0.241	0.957	-1.079	0.008	-0.838	0.015	-0.959	0.001	YES
50	206697_s_at	1.057	0.952	2.333	0.003	1.277	0.069	1.805	0.001	YES
51	206655_s_at	-0.075	0.993	1.125	0.023	1.200	0.005	1.163	0.001	NO
52	208470_s_at	1.178	0.952	2.537	0.003	1.359	0.079	1.948	0.001	NO
53	220001_at	0.443	0.952	1.412	0.006	0.969	0.026	1.191	0.001	NO
54	206209_s_at	0.600	0.952	1.305	0.003	0.705	0.077	1.005	0.001	NO
55	227297_at	0.273	0.957	1.188	0.008	0.915	0.017	1.051	0.001	NO
56	238983_at	0.632	0.952	1.238	0.003	0.606	0.113	0.922	0.001	NO

N°	Probeset	Mod - Mil		Sev -Mil		Sev - Mod		Sev-(Mil & Mod)		Pred. Sign.
		LFC	Adj.P	LFC	Adj.P	LFC	Adj.P	LFC	Adj.P	
57	241652_x_at	0.594	0.952	1.291	0.003	0.697	0.081	0.994	0.001	NO
58	205040_at	0.651	0.952	2.312	0.008	1.661	0.025	1.986	0.001	YES
59	205110_s_at	0.647	0.952	1.709	0.006	1.062	0.054	1.386	0.001	YES
60	205041_s_at	0.777	0.952	2.660	0.008	1.882	0.031	2.271	0.001	NO
61	205627_at	0.413	0.952	1.280	0.008	0.867	0.040	1.074	0.001	NO
62	202252_at	0.296	0.952	1.034	0.008	0.737	0.031	0.885	0.001	NO
63	201061_s_at	0.490	0.952	1.243	0.007	0.753	0.068	0.998	0.002	NO
64	200999_s_at	0.443	0.952	1.100	0.007	0.658	0.072	0.879	0.002	NO
65	209823_x_at	-0.338	0.952	-1.149	0.010	-0.810	0.040	-0.979	0.001	NO
66	229934_at	0.731	0.952	1.249	0.005	0.518	0.232	0.884	0.003	NO
67	220000_at	0.605	0.952	1.429	0.007	0.825	0.086	1.127	0.002	YES
68	211889_x_at	0.637	0.952	1.599	0.008	0.962	0.076	1.280	0.002	YES
69	211883_x_at	0.728	0.952	1.587	0.007	0.859	0.110	1.223	0.002	NO
70	222071_s_at	0.369	0.952	1.088	0.010	0.719	0.058	0.903	0.002	NO
71	202948_at	0.586	0.952	1.445	0.008	0.859	0.080	1.152	0.002	NO
72	216956_s_at	-0.267	0.958	0.791	0.080	1.058	0.008	0.924	0.004	YES
73	211372_s_at	0.843	0.952	1.855	0.007	1.012	0.115	1.433	0.003	NO
74	208168_s_at	0.234	0.959	1.040	0.015	0.806	0.032	0.923	0.002	YES
75	220646_s_at	-0.217	0.965	-1.071	0.017	-0.854	0.030	-0.962	0.002	NO
76	235764_at	0.665	0.952	1.313	0.007	0.648	0.162	0.981	0.003	NO
77	217977_at	0.468	0.952	1.044	0.008	0.575	0.116	0.809	0.003	NO
78	227929_at	0.662	0.952	1.318	0.007	0.656	0.161	0.987	0.003	NO
79	227889_at	0.759	0.952	1.241	0.007	0.483	0.294	0.862	0.005	NO
80	201060_x_at	0.592	0.952	1.242	0.008	0.650	0.144	0.946	0.003	NO
81	205403_at	1.014	0.952	2.014	0.008	1.000	0.165	1.507	0.004	NO
82	204614_at	0.447	0.952	1.108	0.010	0.661	0.096	0.884	0.003	NO
83	206493_at	-0.075	0.994	1.119	0.046	1.194	0.016	1.156	0.004	YES
84	205863_at	0.550	0.952	1.501	0.011	0.951	0.079	1.226	0.003	NO
85	226726_at	0.471	0.952	1.101	0.010	0.630	0.111	0.866	0.003	NO

N°	Probeset	Mod - Mil		Sev -Mil		Sev - Mod		Sev-(Mil & Mod)		Pred. Sign.
		LFC	Adj.P	LFC	Adj.P	LFC	Adj.P	LFC	Adj.P	
86	224480_s_at	0.554	0.952	1.015	0.008	0.461	0.215	0.738	0.005	NO
87	206576_s_at	0.660	0.952	1.616	0.010	0.956	0.101	1.286	0.003	NO
88	240027_at	0.490	0.952	1.121	0.010	0.631	0.123	0.876	0.003	NO
89	222833_at	0.536	0.952	1.025	0.008	0.489	0.194	0.757	0.004	NO
90	215118_s_at	0.070	0.994	-1.230	0.047	-1.300	0.017	-1.265	0.004	YES
91	206177_s_at	0.527	0.957	2.065	0.017	1.539	0.052	1.802	0.003	YES
92	1553518_at	0.187	0.977	1.224	0.024	1.037	0.032	1.130	0.003	NO
93	228648_at	0.956	0.952	1.567	0.008	0.611	0.318	1.089	0.007	NO
94	214465_at	0.353	0.953	1.167	0.016	0.813	0.068	0.990	0.003	NO
95	220615_s_at	0.532	0.952	1.089	0.010	0.556	0.173	0.822	0.005	NO
96	37145_at	-0.191	0.977	-1.232	0.026	-1.041	0.037	-1.136	0.004	NO
97	206157_at	0.177	0.969	1.010	0.024	0.834	0.041	0.922	0.004	NO
98	209369_at	1.156	0.952	2.124	0.009	0.968	0.231	1.546	0.006	YES
99	206522_at	1.104	0.952	1.644	0.008	0.541	0.430	1.093	0.011	NO
100	1553604_at	0.309	0.953	1.103	0.018	0.794	0.065	0.948	0.004	NO
101	213478_at	0.347	0.952	1.075	0.016	0.728	0.077	0.901	0.004	NO
102	205495_s_at	-0.208	0.974	-1.254	0.027	-1.046	0.042	-1.150	0.004	NO
103	1560527_at	0.907	0.952	1.332	0.009	0.425	0.455	0.878	0.013	NO
104	211275_s_at	0.487	0.952	1.126	0.012	0.638	0.140	0.882	0.005	NO
105	210244_at	0.779	0.952	1.778	0.012	0.999	0.145	1.389	0.005	YES
106	232958_at	0.655	0.952	1.233	0.010	0.577	0.227	0.905	0.006	NO
107	200998_s_at	0.321	0.952	1.009	0.018	0.688	0.079	0.848	0.004	NO
108	1553723_at	0.767	0.952	1.380	0.010	0.613	0.258	0.997	0.007	NO
109	218660_at	0.627	0.952	1.210	0.011	0.583	0.219	0.897	0.007	NO
110	219938_s_at	0.452	0.952	1.056	0.014	0.604	0.145	0.830	0.005	NO
111	221485_at	0.570	0.952	1.084	0.011	0.514	0.227	0.799	0.007	NO
112	209238_at	0.610	0.952	1.060	0.010	0.450	0.292	0.755	0.009	NO
113	210004_at	0.436	0.952	1.358	0.019	0.922	0.086	1.140	0.005	YES
114	201110_s_at	-0.277	0.960	0.778	0.122	1.056	0.017	0.917	0.010	NO

N°	Probeset	Mod - Mil		Sev -Mil		Sev - Mod		Sev-(Mil & Mod)		Pred. Sign.
		LFC	Adj.P	LFC	Adj.P	LFC	Adj.P	LFC	Adj.P	
115	203814_s_at	0.377	0.952	1.114	0.019	0.737	0.098	0.925	0.005	NO
116	1562289_at	0.421	0.952	1.251	0.020	0.830	0.098	1.040	0.005	NO
117	209395_at	1.626	0.952	2.468	0.011	0.843	0.436	1.656	0.014	YES
118	206494_s_at	-0.145	0.985	1.069	0.077	1.215	0.024	1.142	0.008	YES
119	201554_x_at	0.452	0.952	1.135	0.017	0.682	0.136	0.908	0.006	NO
120	202391_at	0.634	0.952	1.070	0.012	0.435	0.334	0.752	0.011	NO
121	211734_s_at	-0.325	0.953	-1.045	0.022	-0.720	0.091	-0.883	0.006	YES
122	231886_at	0.802	0.952	1.236	0.012	0.434	0.432	0.835	0.015	NO
123	266_s_at	0.272	0.958	1.022	0.027	0.750	0.081	0.886	0.006	NO
124	227236_at	0.378	0.952	1.010	0.020	0.632	0.132	0.821	0.007	NO
125	220945_x_at	0.982	0.952	1.227	0.014	0.245	0.708	0.736	0.035	NO
126	220404_at	0.684	0.952	1.267	0.015	0.583	0.272	0.925	0.011	NO
127	223836_at	-0.296	0.965	-1.326	0.033	-1.031	0.075	-1.178	0.007	YES
128	220088_at	0.551	0.952	1.025	0.016	0.474	0.276	0.750	0.011	NO
129	206641_at	-0.009	0.999	-1.168	0.062	-1.159	0.045	-1.164	0.009	YES
130	206111_at	0.497	0.952	1.295	0.022	0.798	0.146	1.047	0.008	NO
131	228285_at	0.619	0.952	1.289	0.018	0.670	0.219	0.979	0.010	NO
132	220416_at	0.429	0.952	1.103	0.022	0.673	0.154	0.888	0.009	NO
133	206515_at	1.055	0.952	1.475	0.016	0.420	0.557	0.948	0.025	NO
134	224412_s_at	0.832	0.952	1.209	0.016	0.377	0.513	0.793	0.022	NO
135	220603_s_at	0.605	0.952	1.068	0.017	0.463	0.325	0.765	0.014	NO
136	202499_s_at	0.596	0.952	1.028	0.017	0.433	0.345	0.730	0.015	NO
137	209396_s_at	1.495	0.952	2.210	0.016	0.715	0.497	1.463	0.022	YES
138	227250_at	0.910	0.952	1.291	0.017	0.381	0.548	0.836	0.027	NO
139	230083_at	-0.659	0.952	-1.015	0.017	-0.356	0.460	-0.685	0.021	NO
140	1556185_a_at	0.955	0.952	1.273	0.018	0.318	0.633	0.796	0.034	NO
141	224707_at	0.863	0.952	1.530	0.019	0.667	0.335	1.099	0.015	NO
142	215223_s_at	0.679	0.952	1.158	0.018	0.480	0.364	0.819	0.017	NO
143	229967_at	0.771	0.952	1.409	0.020	0.637	0.313	1.023	0.015	NO
144	211413_s_at	0.323	0.955	1.079	0.033	0.756	0.121	0.917	0.010	NO
145	207890_s_at	0.540	0.952	1.142	0.022	0.603	0.231	0.873	0.013	NO
146	208650_s_at	0.405	0.952	1.164	0.030	0.760	0.146	0.962	0.011	NO
147	225612_s_at	0.605	0.952	1.115	0.020	0.510	0.312	0.813	0.016	NO
148	215783_s_at	0.705	0.952	1.468	0.022	0.763	0.242	1.115	0.013	NO
149	209930_s_at	0.503	0.952	1.009	0.022	0.506	0.262	0.757	0.014	NO
150	226064_s_at	0.572	0.952	1.019	0.020	0.446	0.344	0.733	0.017	NO
151	207674_at	0.688	0.952	1.210	0.020	0.522	0.352	0.866	0.017	NO
152	225987_at	0.670	0.952	1.157	0.020	0.487	0.366	0.822	0.018	NO
153	224327_s_at	0.546	0.952	1.020	0.022	0.475	0.311	0.747	0.016	NO
154	208450_at	0.204	0.984	-1.297	0.114	-1.501	0.048	-1.399	0.016	YES
155	211163_s_at	0.919	0.952	1.254	0.020	0.334	0.615	0.794	0.036	NO
156	204731_at	-0.254	0.962	-1.020	0.042	-0.765	0.109	-0.892	0.012	NO
157	229228_at	0.822	0.952	1.133	0.022	0.310	0.607	0.722	0.038	NO
158	203290_at	0.388	0.981	-1.631	0.220	-2.019	0.107	-1.825	0.050	YES
159	209480_at	-0.050	0.998	0.945	0.602	0.995	0.616	0.970	0.445	YES
160	238439_at	0.735	0.952	1.709	0.040	0.974	0.253	1.341	0.022	YES
161	227474_at	0.399	0.954	-0.278	0.702	-0.677	0.291	-0.477	0.307	YES
162	41469_at	-0.016	0.998	0.862	0.177	0.878	0.162	0.870	0.053	YES
163	203691_at	0.070	0.994	0.921	0.127	0.852	0.154	0.886	0.038	YES
164	218876_at	0.245	0.952	-0.538	0.048	-0.783	0.001	-0.661	0.001	YES
165	201058_s_at	-0.147	0.978	0.848	0.061	0.995	0.013	0.922	0.004	YES
166	229510_at	0.125	0.978	-0.659	0.080	-0.784	0.019	-0.721	0.007	YES
167	202859_x_at	0.330	0.958	1.127	0.046	0.796	0.150	0.961	0.015	YES
168	206632_s_at	0.189	0.986	-0.305	0.761	-0.494	0.628	-0.400	0.552	YES
169	232197_x_at	-0.068	0.989	-0.837	0.021	-0.769	0.016	-0.803	0.002	YES

N°	Probeset	Mod - Mil		Sev -Mil		Sev - Mod		Sev-(Mil & Mod)		Pred. Sign.
		LFC	Adj.P	LFC	Adj.P	LFC	Adj.P	LFC	Adj.P	
170	210321_at	-0.053	0.995	-1.093	0.110	-1.040	0.117	-1.067	0.029	YES
171	224225_s_at	0.216	0.982	0.517	0.552	0.301	0.784	0.409	0.518	YES
172	244523_at	0.280	0.957	0.833	0.065	0.554	0.231	0.693	0.031	YES
173	219519_s_at	-0.538	0.960	-1.011	0.335	-0.473	0.728	-0.742	0.340	YES
174	217148_x_at	-0.092	0.994	-1.168	0.093	-1.075	0.107	-1.121	0.023	YES
175	211748_x_at	-0.321	0.952	-0.885	0.021	-0.564	0.125	-0.724	0.007	YES
176	213506_at	0.571	0.952	0.991	0.064	0.421	0.504	0.706	0.066	YES
177	212592_at	-0.016	0.998	-0.889	0.088	-0.872	0.076	-0.880	0.018	YES
178	234764_x_at	-0.129	0.988	-1.075	0.101	-0.946	0.145	-1.011	0.030	YES
179	239196_at	0.673	0.952	1.350	0.037	0.677	0.326	1.014	0.026	YES
180	219410_at	-0.146	0.967	0.847	0.012	0.994	0.000	0.920	0.000	YES
181	1569110_x_at	0.040	0.996	0.286	0.718	0.246	0.793	0.266	0.637	YES
182	208146_s_at	0.056	0.994	-0.584	0.178	-0.640	0.125	-0.612	0.045	YES
183	201242_s_at	-0.343	0.952	0.406	0.120	0.749	0.001	0.577	0.002	YES
184	221690_s_at	-0.428	0.952	-0.674	0.155	-0.246	0.691	-0.460	0.188	YES
185	206343_s_at	0.199	0.981	-0.211	0.803	-0.409	0.619	-0.310	0.574	YES
186	205678_at	0.241	0.952	0.953	0.000	0.712	0.001	0.832	0.000	YES
187	212187_x_at	-0.279	0.952	-0.820	0.015	-0.541	0.080	-0.680	0.004	YES
188	221530_s_at	0.167	0.963	-0.640	0.056	-0.807	0.005	-0.724	0.002	YES
189	222196_at	-0.147	0.977	-0.412	0.369	-0.265	0.627	-0.338	0.311	YES
190	206647_at	0.142	0.990	0.797	0.322	0.656	0.462	0.727	0.208	YES
191	210262_at	0.116	0.982	0.817	0.063	0.701	0.093	0.759	0.015	YES
192	213537_at	-1.061	0.952	-0.272	0.687	0.789	0.165	0.259	0.592	YES
193	213566_at	-0.005	0.999	-0.594	0.027	-0.589	0.013	-0.591	0.002	YES
194	209290_s_at	0.067	0.988	0.947	0.007	0.881	0.002	0.914	0.000	YES
195	238717_at	-0.003	0.999	-0.443	0.201	-0.440	0.204	-0.442	0.072	YES

References

E1. Templeton KE, Scheltinga S a, Beersma MFC, Kroes ACM, Claas ECJ. Rapid and sensitive method using multiplex real-time PCR for diagnosis of infections by influenza a and influenza B viruses, respiratory syncytial virus, and parainfluenza viruses 1, 2, 3, and 4. *J Clin Microbiol* 2004;42:1564–9.

E2. Ritchie ME, Phipson B, Wu D, Hu Y, Law CW, Shi W, Smyth GK. limma powers differential expression analyses for RNA-sequencing and microarray studies. 2015;1–13.doi:10.1093/nar/gkv007.

E3. Pepe MS. Evaluating technologies for classification and prediction in medicine. Stat Med 2005;24:3687–3696.

E4. Jong VL, Novianti PW, Roes KCB, Eijkemans MJC. Exploring homogeneity of correlation structures of gene expression datasets within and between etiological disease categories. Stat Appl Genet Mol Biol 2014;doi:10.1515/sagmb-2014-0003.

E5. Kim KI, Simon R. Probabilistic classifiers with high-dimensional data. Biostatistics 2011;doi:10.1093/biostatistics/kxq069.

E6. Bernhard Schölkopf and Alexander J. Smola. Learning with Kernels. MIT Press; 2001.

E7. Tibshirani R, Hastie T, Narasimhan B, Chu G. Diagnosis of multiple cancer types by shrunken centroids of gene expression. Proc Natl Acad Sci U S A 2002;doi:10.1073/pnas.082099299.

E8. Breiman L. Random forests. Mach Learn 2001;5–32.doi:10.1023/A:1010933404324.

E9. Mejias A, Dimo B, Suarez NM, Garcia C, Suarez-Arrabal MC, Jartti T, Blankenship D, Jordan-Villegas A, Ardura MI, Xu Z, Banchereau J, Chaussabel D, Ramilo O. Whole Blood Gene Expression Profiles to Assess Pathogenesis and Disease Severity in Infants with Respiratory Syncytial Virus Infection. In: Smyth RL, editor. PLoS Med 2013;10:e1001549.
